# Universal three-dimensional crosslinker for all-photopatterned electronics

**DOI:** 10.1038/s41467-020-15181-4

**Published:** 2020-03-23

**Authors:** Min Je Kim, Myeongjae Lee, Honggi Min, Seunghan Kim, Jeehye Yang, Hyukmin Kweon, Wooseop Lee, Do Hwan Kim, Jong-Ho Choi, Du Yeol Ryu, Moon Sung Kang, BongSoo Kim, Jeong Ho Cho

**Affiliations:** 1 SKKU Advanced Institute of Nanotechnology (SAINT), Sungkyunkwan University (SKKU), Suwon, 16419 Republic of Korea; 2 Department of Chemistry, Korea University, Seoul, 02841 Republic of Korea; 3 Department of Chemical and Biomolecular Engineering, Yonsei University, Seoul, 03722 Republic of Korea; 4 Department of Chemical and Biomolecular Engineering, Sogang University, Seoul, 04107 Republic of Korea; 5 Department of Chemical Engineering, Hanyang University, Seoul, 04763 Republic of Korea; 6 Department of Chemistry, Ulsan National Institute of Science and Technology (UNIST), Ulsan, 44919 Republic of Korea

**Keywords:** Conjugated polymers, Polymer synthesis, Electronic devices

## Abstract

All-solution processing of large-area organic electronics requires multiple steps of patterning and stacking of various device components. Here, we report the fabrication of highly integrated arrays of polymer thin-film transistors and logic gates entirely through a series of solution processes. The fabrication is done using a three-dimensional crosslinker in tetrahedral geometry containing four photocrosslinkable azide moieties, referred to as 4Bx. 4Bx can be mixed with a variety of solution-processable electronic materials (polymer semiconductors, polymer insulators, and metal nanoparticles) and generate crosslinked network under exposure to UV. Fully crosslinked network film can be formed even at an unprecedentedly small loading, which enables preserving the inherent electrical and structural characteristics of host material. Because the crosslinked electronic component layers are strongly resistant to chemical solvents, micropatterning the layers at high resolution as well as stacking the layers on top of each other by series of solution processing steps is possible.

## Abstract (web summary)

To enable the large-area manufacture of solution-processed organic electronics, direct photocrosslinking processes has emerged as a promising technology solution. Here, the authors report an efficient universal crosslinking agent for micropatterning of stacked multi-layered organic electronics.

## Introduction

Solution-processed electronics have been considered a disruptive technology rooted in cost-effective manufacturing^1–8^. The performance of electronic devices based on various solution-processable materials, such as polymer semiconductors, organic small molecules, and nanostructured materials, is being improved continually^6,9–15^. However, fabrication of these devices entirely through a series of solution processes remains highly challenging, as they comprise a stack of fully patterned electronic components, such as electrode layers, charge transporting interlayers, active channel layers, and insulating layers. While various processing methods have been developed for patterning and stacking each of these electronic component layers separately^16–18^, employing multiple techniques/equipment tailored to form respective layers for fabricating devices entirely through a series of solution processes is nontrivial and cost-ineffective. Moreover, new issues arise when a solution-processed layer is exposed to chemicals during the subsequent processing steps. For instance, conventional photolithography requires multiple steps that involve exposure of a given electronic layer to various chemicals, such as photoresist, developer, and etchant^7,16,19^. This means that finding an appropriate orthogonal chemicals that do not damage the underlying electronic layer is critical for successful pattern formation by photolithography^17,20^. The wider spread of this technique for patterning layers of solution-processable materials is thus limited. Inkjet printing and nanoimprinting methods face several difficulties in producing uniform and large-area electronic devices on a large scale^21–26^. They also suffer from the same drawbacks of the solution-processed materials arising from their poor chemical robustness; the prepatterned underlayer is susceptible to the solution-based deposition process of the layer above it. This leads to degradation in the device performance with poor reliability and reproducibility, which is a major obstacle in realizing all-solution-processed electronic devices.

Application of a direct photocrosslinking process to solution-processable materials can be a clever strategy for achieving this goal. Selective exposure to a light source enables patterning of the materials. If the crosslinking density within the materials is high enough, the resulting patterns of the materials would become chemically robust. This would enable stacking of patterns on top of other patterns. Moreover, the thermal stability of the materials would be enhanced because of their crosslinked structure. Accordingly, photocrosslinking agents, such as bifunctional organic azides, have been developed^27–29^, and electronic devices employing these layers have been demonstrated^6,30–37^. To the best of our knowledge, however, patterning and stacking of multiple functional materials by consecutive application of photocrosslinking processes have not yet been attempted. An important remark is that because the crosslinking efficiency per molecule of bifunctional organic azides is as low as 36%^38^, the addition of a large amount of a crosslinking agent is needed to obtain the desired crosslinked structure of the host polymer layer^31,33–36^. In most cases, the use of such a large amount of crosslinking agent leads to a morphological change in the film and deterioration in the electrical and optoelectronic properties of the material. Design of a highly efficient photocrosslinker that can be applied to the patterning process of various solution-processable materials with use of a very low amount is thus highly desirable.

Here, we report a simple processing method for fabricating thin-film patterns of solution-processable semiconductors, gate insulators, and conductors using a highly efficient crosslinking agent that does not yield degradation in the electrical properties of the host materials. A crosslinker, referred to as 4Bx, is structured in three-dimensional tetrahedral geometry containing four photocrosslinkable azide moieties, each of which is placed at the four corners of the molecule. 4Bx is universally applicable to a variety of solution-processable materials containing C–H bonds, including polymer semiconductors, polymeric gate dielectrics, and even conductive metal nanoparticles (NPs) bearing organic surfactants in creating crosslinked network. The given host materials can be successfully photopatterned using a small amount of 4Bx (only 1 wt% for polymer semiconductors and 5 wt% for polymeric gate dielectrics and metal NPs). Moreover, fully patterned polymer thin-film transistors (PTFTs) comprising a stack of these materials are fabricated by all-solution processing. The PTFTs based on photocrosslinked polymer films show equivalent performance and better stability compared with those prepared using polymer films without the crosslinker. Furthermore, we demonstrate the fabrication of NOT, NAND, and NOR logic gates based on arrays of all-photopatterned *p*-type and *n*-type PTFTs.

## Results

### Fabrication of all-photopatterned PTFTs and logic circuits

**Fig. 1:**
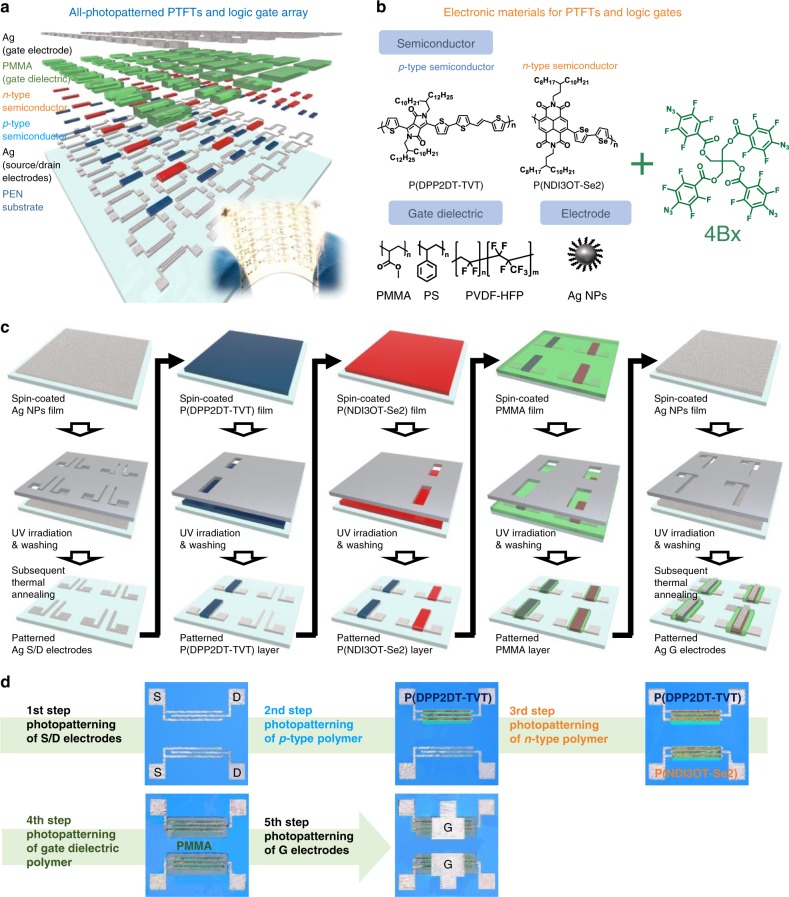
All-solution-processed, all-photopatterned PTFTs and logic circuits. **a** Schematic drawing and photographic image of all-photopatterned PTFTs and logic circuits fabricated on a plastic substrate by patterning of semiconducting channel, gate dielectric, and electrode materials. **b** Chemical structures of electronic materials used in this study. **c** Schematic description of the fabrication processes for *p*- and *n*-type PTFTs through all-photopatterning using 4Bx. **d** Optical microscopy images of the component layers for a PTFT taken after each patterning step.

The schematic drawing of the all-photopatterned PTFTs and logic circuits fabricated on a plastic substrate and the photographic images of the resulting devices are show in Fig. 1a. Chemical structures of the materials used are shown in Fig. 1b. Each device contains a stack composed of Ag NP source (S)/drain (D) electrodes, *p*-type poly((*E*)-2,5-bis(2-decyltetradecyl)-3-(thiophen-2-yl)-6-(5′-(2-(thiophen-2-yl)vinyl)-[2,2′-bithiophen]-5-yl)pyrrolo[3,4-*c*]pyrrole-1,4(2*H*,5*H*)-dione) (P(DPP2DT-TVT)) or *n*-type poly(4-([2,2′-biselenophen]-5-yl)-2,7-bis(3-octyltridecyl)benzo[*lmn*][3,8]phenanthroline-1,3,6,8(2*H*,7*H*)-tetraone) (P(NDI3OT-Se2)) semiconducting channel material, a poly(methyl methacrylate) (PMMA) gate dielectric, and a Ag NP gate electrode. Polystyrene (PS) and poly(vinylidene fluoride-*co*-hexafluoropropylene) (PVDF-HFP) can also be used for the gate dielectric material (see below). Each of these device component layers was photopatterned using a blend solution of the corresponding electronic material and the (2,2-bis(((4-azido-2,3,5,6-tetrafluorobenzoyl)oxy)methyl)propane-1,3-diyl bis(4-azido-2,3,5,6-tetrafluorobenzoate), i.e., 4Bx. 4Bx is structured in three-dimensional tetrahedral geometry containing four photocrosslinkable azide moieties each at the four corners of the molecule. The four azide groups can be activated to generate reactive singlet nitrenes under 254 nm light; the reactive nitrene can undergo the C–H insertion reaction with neighboring alkyl chains to form crosslinked films of the functional materials (see below for further description). Because photocrosslinking reaction can undergo with materials containing alkyl groups, i.e., C–H bonds, 4Bx can be universally applicable to a variety of electronic materials that are processable with common organic solvents; the electronic materials include polymer semiconductors, polymeric gate dielectrics, and even conductive metal NPs bearing organic surfactants. Since the 4Bx molecule has four crosslinkable units, its crosslinking efficiency, defined as the probability of a single crosslinker molecule to successfully undergo the intended C–H insertion reaction, is higher than that of conventional crosslinker with two crosslinkable units. Therefore, only a smaller loading of 4Bx is needed to form crosslinked network of the host material when it is exploited in the photopatterning process (see below for more details)^33,35^. Synthetic details of 4Bx and the polymer semiconductors are provided in Supplementary Methods (Supplementary Figs. 1–21).

The schematic fabrication procedure of *p*- and *n*-type PTFTs by the all-photopatterning process using 4Bx is summarized in Fig. 1c. Typical PTFTs were fabricated in a top-gate bottom-contact (TGBC) configuration on a polyethylene naphthalate (PEN) substrate. The conducting electrodes, semiconducting channel materials, and polymer gate dielectrics were sequentially photopatterned as follows. The first step was the patterning of the Ag NP S/D electrodes. This was done by spin-coating a mixture solution of Ag NPs and 4Bx onto a PEN substrate, followed by selective exposure to UV light (254 nm) through a photomask. The detail photocrosslinking mechanism of Ag NPs is described below. The uncrosslinked regions were removed simply by the solvent used to coat the Ag NPs and 4Bx mixture, and the resulting photopatterned Ag NP film was then thermally annealed. The annealing process was necessary to impart metallic conductivity to the Ag NP patterns. We note that this two-step process, i.e., photopatterning and thermal annealing, allows us to achieve a high resolution patterning of conductive Ag NP patterns. The second step was the patterning of the *p*-type polymer film, which was achieved by spin-casting a mixture solution of the *p*-type polymer and 4Bx, exposing the desired area to UV through a photomask, and then washing out the uncrosslinked polymer regions. As long as a solvent can dissolve the pristine semiconducting polymer well, it could serve as a good developing solvent (Supplementary Fig. 22). The third step was the photopatterning of the *n*-type polymer film by the same method as that was used in the second step for patterning the *p*-type polymer film. The consecutive application of photopatterning process was only possible due to the structural robustness of the underlying crosslinked *p*-type polymer film. The fourth step was the photopatterning of the polymer gate dielectric layer in the same way by spin-coating of a mixture solution of PMMA insulator and 4Bx. In the fifth step, Ag NP films were again photopatterned onto the *p*- and *n*-channel regions. Optical microscopy images of the patterned PTFT device layers after each photopatterning step are shown in Fig. 1d. We found that all the crosslinked thin films showed improved chemical resistance, and thus, the solvent used in each step for both spin-coating and washing did not damage the underlying crosslinked layer. This implies that stacking and patterning multiple layers of functional materials is possible by the described steps. We emphasize that our study demonstrates the fabrication of all-photopatterned, all-solution-processed PTFTs, which was achieved following a single processing protocol i.e., the photopatterning based on 4Bx for different functional components of devices.

### Characterization of crosslinked and photopatterned electronic components

**Fig. 2:**
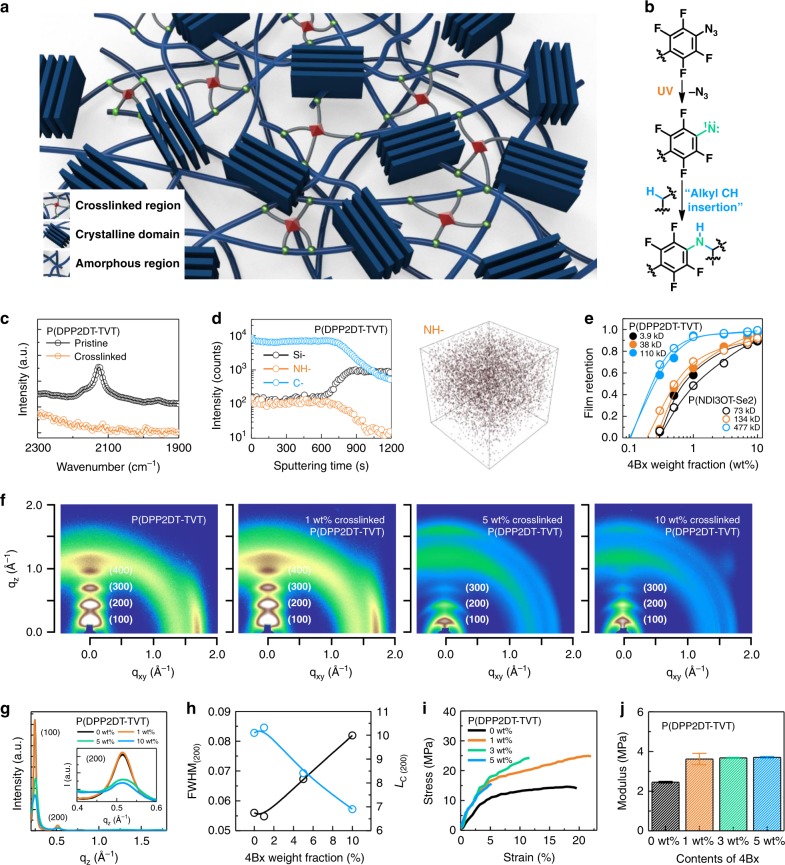
Structural characterization of crosslinked P(DPP2DT-TVT). **a** Schematic illustration of a semiconducting polymer in its crosslinked state formed by 4Bx. **b** The key chemical reaction involved for the crosslinking. **c** FTIR spectra of P(DPP2DT-TVT) films before and after being crosslinked. **d** TOF-SIMS spectra of crosslinked P(DPP2DT-TVT) film monitored as a function of sputtering time. **e** Film retention characteristics of P(DPP2DT-TVT) and P(NDI3OT-Se2) formed with different contents of 4Bx. **f** 2D GIXD patterns of a series of P(DPP2DT-TVT) films using different loadings of 4Bx. **g** The line-cut diffraction profiles of the diffractograms for P(DPP2DT-TVT) films along the of out-of-plane direction. The inset is an enlarged view of the plot near the (200) peak. **h** Summary of FWHM and characteristic coherence length (*L*_C_) of the out-of-plane (200) peak for P(DPP2DT-TVT) films crosslinked using different loadings of 4Bx. **i** The stress–strain curves of a series of P(DPP2DT-TVT) films crosslinked using different loadings of 4Bx (0, 1, 3, and 5 wt%). **j** Tensile modulus of a series of P(DPP2DT-TVT) films crosslinked using different loadings of 4Bx (0, 1, 3, and 5 wt%).

Figure 2a, b shows a schematic illustration of semiconducting polymer film photocrosslinked by 4Bx and the underlying chemical reaction. When a semiconducting polymer film containing 4Bx is exposed to UV light, the photolysis of the azide groups in 4Bx generates highly reactive singlet nitrene (-^1^N) along with the evolution of inert nitrogen gas^33,35,38,39^. The dominant reaction between the semiconducting polymer and 4Bx is the insertion of the singlet nitrene into the C–H bond within the alkyl chains of the neighboring polymers^35,38^, which results in the three-dimensionally networked structure. The photochemical reaction of the 4Bx crosslinker inside the P(DPP2DT-TVT) and P(NDI3OT-Se2) films was confirmed by Fourier transform infrared (FTIR) spectroscopy before and after the films were exposed to UV light, as shown in Fig. 2c and Supplementary Fig. 23a, respectively. The characteristic vibration peak of the azide groups in 4Bx at 2125 cm^−1^ disappeared entirely after the films were exposed to UV. The distributions of the photocrosslinked positions could be identified by time-of-flight secondary ion mass spectrometry (TOF-SIMS). The depth profiles were obtained as a function of sputtering time for photocrosslinked polymer films coated on Si wafers. For both polymers, 4Bx molecules in their crosslinked state, yielding signals of –NH component, were found to be distributed uniformly over the film thickness (Fig. 2d and Supplementary Fig. 24a). In addition, both the pristine and crosslinked P(DPP2DT-TVT) and P(NDI3OT-Se2) films showed similar surface morphologies (see the AFM images of these polymer films in Supplementary Fig. 25).

The high crosslinking efficiency of 4Bx is a key factor allowing only a small loading of the material to produce crosslinked state of the semiconducting polymer films; this is critical for the patterning polymer films while maintaining their optical and electrical characteristics. The photocrosslinking efficiency of 4Bx crosslinker for P(DPP2DT-TVT) and P(NDI3OT-Se2) was evaluated indirectly by the film retention, defined as the relative change in the optical absorption at wavelength of 817 and 735 nm, respectively, upon rinsing with chloroform where the light absorption is most intense for the respective polymers (Supplementary Fig. 26). If these polymer films are not crosslinked effectively, they would be removed during the rinsing process and thus the film retention would be low. These values are plotted as a function of the weight fraction of 4Bx added into the film (Fig. 2e). The optical density of the films (that is proportional to the film thickness) prepared from higher contents of 4Bx could be preserved more effectively, and the polymer films prepared from batches with the highest molecular weight could be preserved using as little as 1 wt% of the crosslinker (the contents of crosslinker in mol% relative to the moles of the polymer repeating units are provided as well in Supplementary Table 1), which is the lowest weight fraction reported thus far for crosslinking of polymer semiconductors^31,33–36^. The successful production of a fully crosslinked state using only 1 wt% of 4Bx has a significant meaning, because the use of such a small amount of crosslinker would suppress the side effects caused by the photocrosslinking step on the chemical, physical, and electrical properties of semiconducting polymers. Therefore, in the follow-up experiments, 1 wt% of 4Bx was utilized, if not noted.

For the structural characterization, two-dimensional grazing incidence X-ray diffraction (2D GIXD) analysis was carried out for photocrosslinked P(DPP2DT-TVT) and P(NDI3OT-Se2) films prepared with different loading of 4Bx (0, 1, 5, and 10 wt%). Figure 2f shows 2D diffractograms of the series of P(DPP2DT-TVT) films. The series of P(DPP2DT-TVT) films showed ordered lamellar peaks in the out-of-plane direction and a π‒π stacking peak in the in-plane direction, indicating that P(DPP2DT-TVT) is oriented dominantly in an edge-on structure. Figure 2g shows the line-cut 1D profiles of the diffractograms along the out-of-plane direction. The peak position observed from the profiles reveals the average spacing between the ordered units in semicrystalline polymer film. The peak intensity allows comparing the crystallinity of semicrystalline films with equivalent scattering volume. The broadness of the peaks (represented by the FWHM) allows estimating the characteristic coherence length (*L*_C_) that scales with the average crystalline domain size of the semicrystalline film. Structural parameters of the films extracted from the diffractograms are summarized in Supplementary Table 2. When high contents of 4Bx (5 and 10 wt%) were used, the position of the diffraction peaks did not change compared with that from pristine P(DPP2DT-TVT) film, but noticeable reduction in the peak intensity and broadening of the peaks were observed (Fig. 2g, h). When P(DPP2DT-TVT) was photocrosslinked using 1 wt% of 4Bx, on the other hand, the position, intensity, and broadness of the peaks and the associated structural parameters of the films were nearly preserved, compared with those obtained from pristine P(DPP2DT-TVT) film with equivalent thickness. This result indicates that only a minute change (if any) in the molecular packing of P(DPP2DT-TVT) was induced, when 1 wt% of 4Bx was used. Note that this condition of using 1 wt% of 4Bx corresponds to the optimal condition that allows photopatterning P(DPP2DT-TVT) with minimal loss in its electrical properties, which will be described below. In sum, these results indicate that the crystallinity of the films is weakened when P(DPP2DT-TVT) is cast together with a large amount of 4Bx and become crosslinked after exposure to UV. The results also indicate that the inherent crystallinity of the P(DPP2DT-TVT) films can be preserved by using a small amount of 4Bx due to its high crosslinking efficiency. Despite the structures of the films not being altered noticeably, recall that the crosslinked P(DPP2DT-TVT) films using only 1 wt% of 4Bx showed excellent retention characteristics against chemical solvents. A consistent behavior was observed for P(NDI3OT-Se2) films (Supplementary Fig. 27).

We further examine the mechanical properties of the photocrosslinked P(DPP2DT-TVT) and P(NDI3OT-Se2) films by monitoring their strain–stress relation. This was done by employing the film-on-water apparatus on a pseudofreestanding polymer thin films floated on water^40,41^. Figure 2i shows the stress–strain relations of a series of P(DPP2DT-TVT) films prepared with different loadings of 4Bx (0, 1, 3, and 5 wt%). With increasing the contents of 4Bx, the film became more brittle and its elastic modulus increased, which is another indication that crosslinked polymer structure is developed by using 4Bx (Fig. 2j). Consistent results were also observed from P(NDI3OT-Se2) films (Supplementary Fig. 28). In addition, an increase in the glass transition temperature (*T*_g_) by 8.5 °C was observed for P(DPP2DT-TVT) films when they were crosslinked using 5 wt% of 4Bx (Supplementary Fig. 29).

**Fig. 3:**
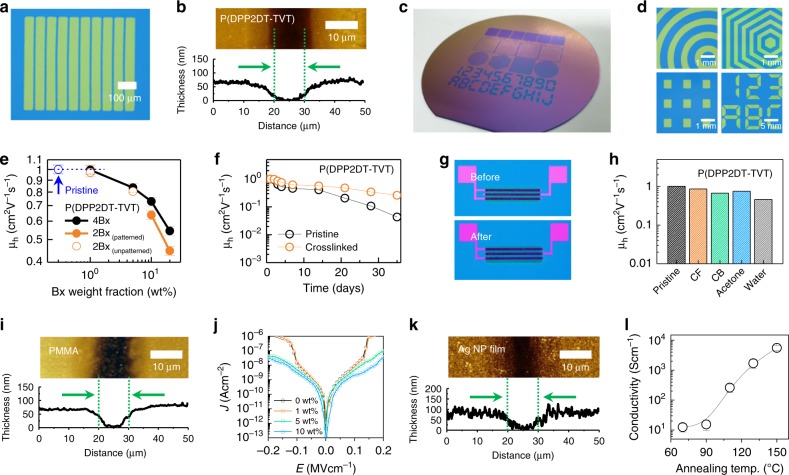
Characterization of electronic components photopatterned using 4Bx. **a** OM image of photopatterned P(DPP2DT-TVT) films with a spacing of 10 µm. **b** AFM image and height profile of photopatterned P(DPP2DT-TVT) films. **c** Photograph of a 6-in. Si/SiO_2_ wafer with P(DPP2DT-TVT) patterns. The polymer film was cast onto the wafer by bar-coating process before the photopatterning process. **d** Optical microscopy top-view image of the patterns of P(DPP2DT-TVT) shown in **c**. **e** Hole mobilities for PTFTs based on photocrosslinked P(DPP2DT-TVT) channel plotted as a function of the loading of the two crosslinkers (4Bx and 2Bx). Note that the data in open circle are obtained from a photocrosslinked P(DPP2DT-TVT) channel prepared using 1 wt% of 2Bx. The resulting films did not exhibit good retention characteristics, and thus the films prepared using 1 wt% of 2Bx cannot be utilized for the patterning process. **f** Comparison of the hole mobilities over time for pristine and crosslinked P(DPP2DT-TVT) PTFTs. **g** OM images of a PTFT based on photopatterned P(DPP2DT-TVT) before and after being soaked in CF. **h** Hole mobilities of PTFTs based on photopatterned P(DPP2DT-TVT) before and after being soaked in various solvents. **i** AFM image and height profile of photopatterned PMMA film. **j** Current density–electric field plot of PMMA films crosslinked using different loadings of 4Bx. **k** AFM image and height profile of photopatterned Ag NP film. **l** Electrical conductivity of Ag NP films annealed at different temperatures.

The capability to form patterns was then examined upon varying the weight fraction of 4Bx included in the film; the procedure includes formation of a polymer film containing 4Bx, selective exposure of the film to UV through a photomask, and removal of the uncrosslinked region of the film using organic solvent. For comparison, not only the 4Bx but also the sister crosslinker, ethane-1,2-diyl bis(4-azido-2,3,5,6-tetrafluorobenzoate) (2Bx) comprising only two crosslinkable units at the termini of the molecules, was evaluated. High-resolution patterns of P(DPP2DT-TVT) could be achieved using only 1 wt% of 4Bx (Supplementary Fig. 30a), thanks to the high crosslinking efficiency of the molecule. By contrast, even the use of 7 wt% of 2Bx was not sufficient to form sharp patterns of P(DPP2DT-TVT)^31,33–36^. Multiple strips of photopatterned P(DPP2DT-TVT) films with a spacing of 10 µm could also be obtained successfully using 1 wt% of 4Bx (Fig. 3a). An AFM image and height profile of the photopatterned P(DPP2DT-TVT) film are shown in Fig. 3b. These films and the images in Fig. 3a, b are obtained from films originally cast by spin-coating method prior to the photopatterning process. We note that the photopatterning process can also be applied to films formed from simple bar-coating method that is highly desirable for scale-up. Figure 3c and Supplementary Fig. 31 show a photograph of a 6-in. Si/SiO_2_ wafer with P(DPP2DT-TVT) patterns, which were cast onto the substrate by bar-coating process. Figure 3d shows the optical microscopy images of the resulting patterns. The results demonstrate that the method is highly adaptable to high-throughput manufacturing.

The electrical properties and stabilities of photocrosslinked semiconducting polymer films were examined using a PTFT device testbed in the bottom-gate top-contact (BGTC) configuration. For the testbed, only the semiconducting polymer layers were photopatterned. Au contacts prepared from thermal evaporation were employed as the source/drain electrodes and an octadecyltrichlorosilane (ODTS)-treated SiO_2_/Si wafer was used as the gate/gate dielectric. Carrier mobility (*μ*) of the polymer films was calculated in the saturation regime of the transistor operation according to the equation *I*_D_ = *C*_S_ · *μ* · *W* · (*V*_G_ − *V*_TH_)^2^/2*L*^42–44^, where *C*_S_ is the specific capacitance of the gate dielectric; *V*_TH_ is the threshold voltage; and *W* and *L* are the channel width and length, respectively. The values of *μ* for P(DPP2DT-TVT) channels photocrosslinked with 4Bx and 2Bx are plotted in Fig. 3e as a function of the crosslinker weight fraction. The hole mobilities decreased with increasing the weight fraction of crosslinkers. The poorer crystallinity of the film as well as the insertion of a larger amount of insulating crosslinker molecule would interrupt charge transport in the photocrosslinked films^45^. It is noteworthy that P(DPP2DT-TVT) films that are crosslinked only with 1 wt% of 4Bx showed nearly the same hole mobility as those of pristine P(DPP2DT-TVT) films. This can be well explained with the fact that photocrosslinking with the use of 1 wt% 4Bx did not make any morphological changes in the film structure as explained above. Actually, the hole mobility of P(DPP2DT-TVT) films crosslinked with 1 wt% of 2Bx showed a similar value. However, unlike 4Bx, using only 1 wt% of 2Bx did not form P(DPP2DT-TVT) films with good film retention characteristics against chloroform, which is mandatory for the photopatterning process. Patterning is only possible when crosslinking of polymers is done sufficiently so that the resulting film gains good retention characteristics against subsequent developing processes (Supplementary Fig. 30a). For 2Bx, >7 wt% was required to form chemically robust P(DPP2DT-TVT) films, which, in turn, lead to the severe reduction in hole mobility. A similar trend was also observed for *n*-type PTFTs based on the P(NDI3OT-Se2) film photopatterned by 4Bx (Supplementary Fig. 32). These observations altogether highlight that the use of 4Bx does not lead to suppression in the electrical properties of the host semiconducting polymers even after photopatterning.

In addition, the stabilities of the pristine and crosslinked PTFTs were compared. First, the shelf life stability of the PTFTs was examined by monitoring the mobility of the devices over time. It was observed that the initial hole mobility was better preserved for the crosslinked films than for the pristine films (Fig. 3f and Supplementary Fig. 33). The higher shelf life stability of PTFTs based on crosslinked polymer channel is perhaps due to the slower diffusion of oxygen or water molecules within the photocrosslinked polymer film. Second, the chemical stability of the PTFTs was examined. This was done by comparing the performances of photopatterned PTFTs based on P(DPP2DT-TVT) and P(NDI3OT-Se2) channels crosslinked with 1 wt% of 4Bx with those obtained after soaking the devices into four different solvents (CF, chlorobenzene, acetone, and deionized water) for 5 min. No apparent change was observed from optical microscopy images for the photocrosslinked P(DPP2DT-TVT) PTFTs (Fig. 3g), and their carrier mobilities were only affected slightly (Fig. 3h). Similar results were obtained after solvent-soaking the photocrosslinked P(NDI3OT-Se2) PTFTs (Supplementary Fig. 34). In contrast, the carrier mobilities of the PTFTs based on uncrosslinked, pristine polymer channels decreased dramatically after solvent-soaking (Supplementary Fig. 35). These results indicate that electrical properties of the photocrosslinked polymer channels can be preserved during the developing process, i.e., the step of removing the uncrosslinked regions of the polymer thin films using chemical solvents during the patterning process.

4Bx was also used to photopattern insulating polymers, such as PMMA, PS, and PVDF-HFP (Supplementary Fig. 30b), which can serve as the gate dielectric layer of PTFTs. Consistent with the crosslinking done for the semiconducting polymers, these insulating polymers could be photocrosslinked according to the C–H insertion reaction of the UV-activated nitrene groups of 4Bx uniformly across the entire thickness of the film (Supplementary Figs. 23b, c and 24b). The AFM image and height profile of a photopatterned PMMA film are shown in Fig. 3i, which demonstrates that patterns with a feature size < 10 µm can be formed using 4Bx. The frequency-dependent capacitance, representing the dielectric properties of an insulator, was identical for the pristine and crosslinked PMMA films (Supplementary Fig. 36). This indicates that the photopatterning process did not cause a series adverse effect on the capacitive properties of the material. The current density (*J*)–electric field (*E*) curves for the PMMA films (thickness = 480 nm) in a metal/insulator/metal (MIM) device geometry were also obtained, which were incorporated with various amounts of the 4Bx (0–10 wt%). Using a small amount of 4Bx (<5 wt%) resulted in a large leakage current for the crosslinked PMMA films at an electric field of >0.1 MV cm^−1^ (Fig. 3j). The leakage current was significantly suppressed when >5 wt% of 4Bx was used. This result suggests that the leakage current through the PMMA films can be effectively reduced upon the formation of a tight structural network via a high degree of crosslinking. Finally, 4Bx could be used to photopattern Ag NPs. The Ag NPs are composed of core Ag atoms surrounded with aliphatic surfactants (Supplementary Fig. 37), and the aliphatic surfactants can undergo the crosslinking reaction with the use of 4Bx under UV. Films of Ag NPs could thus be photopatterned by using 5 wt% of 4Bx with a narrow feature size (~10 µm) (Fig. 3k). Once the Ag NP films were patterned, they were annealed at different temperatures (70–150 °C). Conductivity as high as 5500 S cm^−1^ was obtained from Ag NP films annealed at 150 °C (Fig. 3l). This conductivity is high enough for the Ag NP films to be used as the electrodes for PTFTs, while the annealing temperature is low enough for the films to be processed onto a flexible plastic substrate. Overall, we demonstrated that 4Bx can be added to the entire electronic components of PTFTs, i.e., *n-* or *p-*type channel layer, gate dielectric layer, and electrodes, allowing photopatterning each of these component layers.

### Electrical characteristics of all-photopatterned PTFTs and logic circuits

**Fig. 4:**
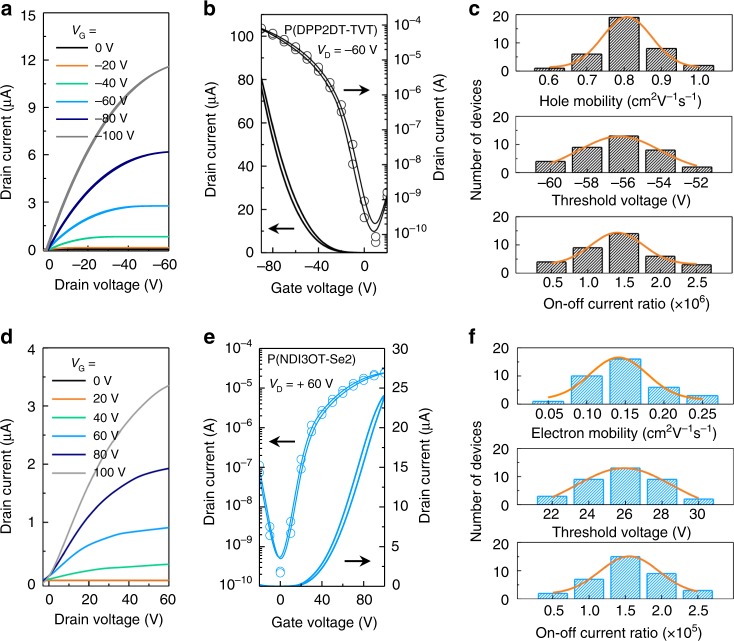
Electrical characteristics of all-photopatterned PTFTs. **a** Output and **b** transfer characteristics of PTFTs based on P(DPP2DT-TVT) crosslinked using 1 wt% of 4Bx. **c** Summary of hole mobility (top), threshold voltage (middle), and on–off current ratio (bottom) of 36 P(DPP2DT-TVT) PTFTs. **d** Output and **e** transfer characteristics of PTFTs based on P(NDI3OT-Se2) crosslinked using 1 wt% of 4Bx. **f** Summary of electron mobility (top), threshold voltage (middle), and on–off current ratio (bottom) of 36 P(NDI3OT-Se2) PTFTs.

Exploiting the optimized processes developed for each of these components, all-photopatterning of PFTFs and logic circuits based on these devices was conducted, according to the protocol described in Fig. 1c. The output characteristics [drain current (*I*_D_) versus drain voltage (*V*_D_)] of a single all-photopatterned *p*-type PTFT based on P(DPP2DT-TVT) channel, PMMA gate dielectric (specific capacitance = 5 nF cm^−2^), and Ag NP electrodes at six different gate voltages (*V*_G_s) are shown in Fig. 4a. The curves demonstrate the gate modulation with a linear behavior at low-*V*_D_ region and a saturation behavior at high-*V*_D_ region. The observation of the linear behavior at low-*V*_D_ region indicates that an Ohmic contact is formed between the P(DPP2DT-TVT) channel and Ag NP electrodes. The transfer characteristics (*I*_D_ versus *V*_G_) of the *p*-type PTFT at a fixed *V*_D_ of −60 V are shown in Fig. 4b. |*I*_D_| increased with increasing *V*_G_ negatively, which is a typical signature of *p*-type transistor operation. No obvious hysteresis was observed between the forward and reverse sweeps, implying a small density of trap sites presenting at the semiconductor/dielectric interface. The electrical properties of 36 all-photopatterned P(DPP2DT-TVT) PTFTs fabricated in a single batch of experiment are summarized in Fig. 4c. The average hole mobility was estimated to be 0.81 (±0.18) cm^2^ V^−1^ s^−1^. It is noteworthy that a maximum mobility of 1.03 cm^2^ V^−1^ s^−1^ was obtained even though all electronic components were photopatterned. Average on–off current ratio was 1.6 (±0.8) × 10^6^. Average *V*_TH_ was −56 (±4) V. Average turn-on voltage (*V*_on_), defined as the gate voltage yielding the onset of the channel current was +7.7 (±0.5) V. Nearly equivalent device performance was obtained from all-photopatterned P(DPP2DT-TVT) TFTs using the same materials but in the BGTC configuration (Supplementary Fig. 38). Furthermore, a polymer gate dielectric with higher capacitance, e.g., a photopatterned PVDF-HFP layer (specific capacitance = 11.1 nF cm^−2^), could be employed for the device, demonstrating the reduction of the operation voltages. The average *V*_TH_ and *V*_on_ for the all-photopatterned TFTs with PVDF-HFP gate dielectric were reduced to −11 and +0.7 V, respectively, and these devices could operate at reduced gate voltages below 20 V (Supplementary Fig. 39).

The *n*-type PTFTs were fabricated using P(NDI3OT-Se2), PMMA gate dielectric, and Ag NP electrodes. The output characteristics of a single P(NDI3OT-Se2) TFT show an increase in *I*_D_ with a positive increase in *V*_G_, which is a signature of *n*-type transistor operation (Fig. 4d). The representative transfer characteristic of the P(NDI3OT-Se2) TFT at *V*_D_ = +60 V is shown in Fig. 4e. The histograms obtained from 36 all-photopatterned *n*-type PTFTs reveal average electron mobility of 0.15 (±0.09) cm^2^ V^−1^ s^−1^, *V*_TH_ of 26 (±4) V, *V*_on_ of −0.6 (±0.4) V, and on–off current ratio of 1.5 (±0.9) × 10^5^. Consistent with the *p*-type devices, the all-photopatterned *n*-type PTFTs employing a high capacitance PVDF-HFP gate dielectric layer operated at reduced gate voltages below 20 V (Supplementary Fig. 39 and Supplementary Table 3). PTFTs based on six other semiconducting polymers were fabricated by the same method using 1 wt% of 4Bx (Supplementary Fig. 40), and their device characteristics are summarized in Table 1. These data demonstrate that our photopatterning approach using 4Bx is generally applicable to a variety of polymer semiconductors.

**Table 1:** Summary of electrical characteristics of 36 all-solution-processed, all-photopatterned PTFTs.

Semiconducting polymer	Carrier mobility (cm^2^ V^−1^ s^−1^)	On–off current ratio	Threshold voltage
P(DPP2DT-TVT)	0.81 (±0.18) **1.03**	1.6 (±0.8) × 10^6^	−56 (±4) V
P(DPP2DT-F2T2)	0.72 (±0.21) **0.94**	2.5 (±0.5) × 10^6^	−74 (±5) V
P(DPP2DT-T2)	0.25 (±0.09) **0.35**	1.4 (±1.1) × 10^6^	−60 (±3) V
PTB7-Th	0.005 (±0.001) **0.007**	4.1 (±1.8) × 10^5^	−42 (±6) V
P(NDI3OT-Se2)	0.15 (±0.09) **0.24**	1.5 (±0.9) × 10^5^	26 (±4) V
P(NDI2OD-F2T2)	0.10 (±0.03) **0.13**	2.1 (±1.1) × 10^6^	47 (±7) V
P(NDI2OD-Se2)	0.14 (±0.05) **0.19**	6.1 (±0.4) × 10^5^	74 (±8) V
P(NDI3OT-Se)	0.14 (±0.07) **0.21**	2.8 (±0.8) × 10^5^	28 (±4) V

Bold numbers indicate the maximum mobility values obtained from each series.

**Fig. 5:**
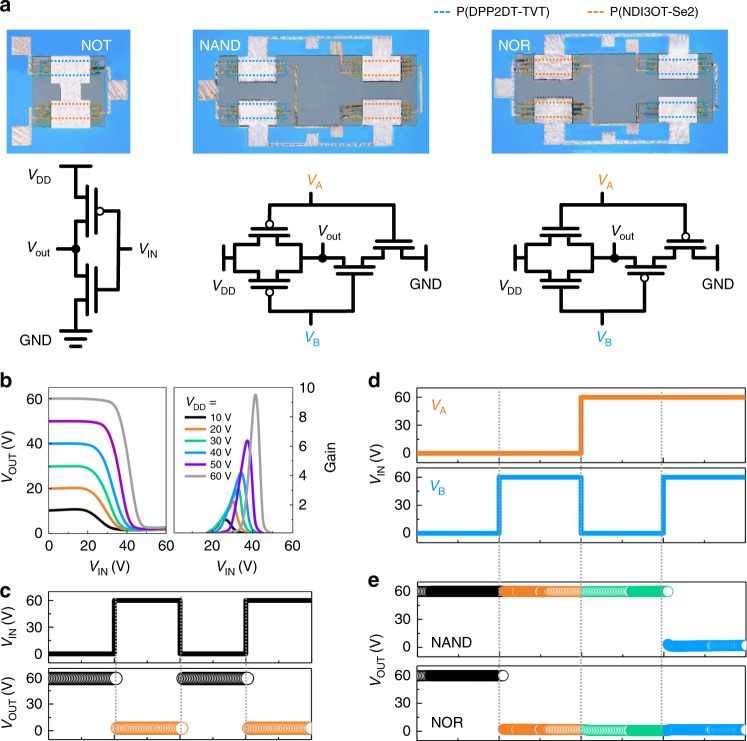
Electrical characteristics of all-photopatterned logic circuits. **a** OM images and schematic circuit diagrams of NOT, NAND, and NOR logic gates based on all-photopatterned *p*-type P(DPP2DT-TVT) and *n*-type P(NDI3OT-Se2) PTFTs. **b** Voltage transfer curves and signal inverter gain of NOT gate. **c** Response characteristics of NOT gate. **d** Four possible logic combinations: (0, 0), (1, 0), (0, 1), and (1, 1). **e** Output voltages of NAND and NOR gates for the four logic combinations in **d**.

Fabrication of all-photopatterned *p*-type and *n*-type PTFTs with reliable operation enabled assembling these devices into functional complementary logic circuits, as shown in Fig. 5^46,47^. OM images and the corresponding circuit diagrams of these all-photopatterned complementary logic circuits, i.e., NOT, NAND, and NOR logic gates, are shown in Fig. 5a. First, the NOT gate was fabricated by connecting a *p*-type and an *n*-type PTFTs in series. The voltage transfer characteristics of the NOT gate at various supply voltages (*V*_DD_s) of 10, 20, 30, 40, 50, and 60 V are shown in Fig. 5b. When the input voltage (*V*_IN_) was in the logic state “0” (*V*_IN_ = 0 V), the output voltage (*V*_OUT_) yielded the logic state “1” (*V*_OUT_ = *V*_DD_). Signal inversion was clearly observed with a positive increase in *V*_IN_ such that *V*_OUT_ decreased to 0 V (logic state “0”). The signal inverter gain of the NOT gate, defined as |*dV*_OUT_/*dV*_IN_|, was determined to be ~10 at *V*_DD_ = 60 V. The output signal obtained at *V*_DD_ = 60 V is shown in Fig. 5c. *V*_OUT_ switched from the “1” state to the “0” state when *V*_IN_ was switched from the “0” state to the “1” state, and vice versa. More complicated logic circuits, such as NAND and NOR gates, could also be fabricated by assembling four PTFTs. The NAND gate was fabricated by connecting two *n*-type PTFTs in series and two *p*-type PTFTs in parallel, whereas the NOR gate was fabricated by connecting two *n*-type PTFTs in parallel and two *p-*type PTFTs in series. The four possible input logic combinations, (0, 0), (1, 0), (0, 1), and (1, 1), are shown in Fig. 5d; here, the input voltages *V*_A_ and *V*_B_ were switched between 0 and 60 V at a constant *V*_DD_ of +60 V. As observed in Fig. 5e, both the NAND gate and the NOR gate showed different output signals at different combinations of input signals. For the NAND gate, *V*_OUT_ was in the logic state “0” only for the (1, 1) logic combination, and it was in the logic state “1” for the other three logic combinations, i.e., (0, 0), (1, 0), and (0, 1). For the NOR gate, on the other hand, *V*_OUT_ was in the logic state “1” only for the (0, 0) logic combination, and it was in the logic state “0” for the other three logic combinations, i.e., (1, 0), (0, 1), and (1, 1). Both these complementary logic gates showed ideal rail-to-rail output voltages in the corresponding input states. These results demonstrate the logic circuits prepared from all-solution, all-photopatterned process which should be expendable to even a complicated circuitry design.

## Discussion

We herein described a photopatterning approach based on 4Bx with high crosslinking efficiency for realizing all-solution-processed organic electronic devices. Different types of electronic materials, i.e., semiconducting polymers, insulating polymers, and metal NPs, were successfully photocrosslinked using a very small amount of 4Bx. Semiconducting polymers could be successfully photocrosslinked using only 1 wt% of 4Bx without altering their intrinsic crystallinity and charge transport properties. Moreover, the good solvent resistance of the photocrosslinked films resulted in high-resolution polymer micropatterns. This pivotal feature facilitated multistacking of the photopatterned electronic component layers and the fabrication of an array of all-solution-processed, all-photopatterned PTFT devices. NOT, NAND, and NOR logic gates could be successfully fabricated by all-photopatterning *p*-type and *n*-type PTFTs. Overall, this work demonstrates an effective route to all-solution-processed organic electronic devices based on a single fabrication protocol.

## Methods

### Film characterizations

The photochemical reaction of 4Bx in the electronic component layers was confirmed by FTIR spectroscopy (Bruker IFS-66/S, TENSOR27). The spatial distribution of the crosslinking points was identified by TOF-SIMS (ION-TOF, Germany). Bi_3_^2+^ (30 keV) was used as the primary ion source in TOF-SIMS. Negative secondary ions from an area of 100 × 100 μm^2^ were detected. Sputter etching was performed over an area of 400 × 400 μm^2^ using an Ar gas cluster ion beam with an acceleration voltage of 2.5 keV. The currents of the primary ion beam and Ar gas cluster ion beam were 0.5 pA and 0.9 nA, respectively, for the polymer films and 0.9 pA and 40.0 nA, respectively, for the Ag NP film. The crystalline structures of the semiconducting polymer films were examined by 2D GIXD at the 9A beamline of the Pohang Light Source II, Republic of Korea. Tapping-mode AFM images were obtained using Multimode-N3-AM (Bruker/Veeco/Digital Instruments). UV–visible absorption spectra were recorded on the UV-3600 spectrophotometer (Shimadzu). The glass transition temperature was measured by spectroscopic ellipsometry (SEMG-1000, Nano-view) with a thermal stage (2 °C per min) under vacuum. The electrical properties of the PTFTs were measured at room temperature under dark using a Keithley 4200 semiconductor characterization system. Specific capacitances of the polymer gate dielectrics were measured in the MIM device geometry using the Agilent E-4980a LCR meter in the frequency range of 100 Hz to 1 MHz. For the fabrication of capacitors, PMMA was deposited onto a heavily doped Si substrate, and this was followed by the thermal evaporation of top Au electrodes (~40 nm). The area of each Au electrode was 500 μm^2^. Crosslinked Ag NP film was sintered at different temperatures (70–150 °C) in a vacuum oven (~10^−3^ Torr) for 8 h. The electrical conductivity of the sintered Ag NP film was measured by the four-point probe method (Keithley 2182A and 6221).

### Fabrication of BGTC PTFT testbed devices

For testing of the electrical properties and stabilities of photocrosslinked polymers, BGTC PTFTs were fabricated on a heavily *n*-doped Si wafer having a thermally grown 300-nm-thick SiO_2_ layer. The SiO_2_/Si substrate was cleaned by ultrasonication with acetone, isopropanol, and deionized water in sequence for 10 min each. The SiO_2_ surface was treated with ODTS (Gelest, Inc.) to prevent charge trapping by silanol groups on the SiO_2_ surface. Blend solutions of *p*-type P(DPP2DT-TVT) or *n*-type P(NDI3OT-Se2) polymer and various amounts of crosslinker (4Bx or 2Bx) with a concentration of 5 mg mL^−1^ were spin-coated onto the ODTS-treated SiO_2_/Si substrate. After photopatterning (254 nm, 1000 W cm^−2^, 30 s) using a shadow mask inside a N_2_-filled glove box, the photopatterned polymer films were stored for 4 h to remove the residual solvent. Finally, 50-nm-thick Au S/D electrodes were thermally deposited onto the photocrosslinked semiconducting polymer films through a shadow mask. The channel length (*L*) and width (*W*) of the S/D electrodes were 100 and 800 µm, respectively.

### Fabrication of TGBC all-photopatterned PTFTs and logic gate array

PEN (Teijin DuPont Films, Teonex Q65HA) was used as the plastic substrate after cleaning by ultrasonication with acetone, isopropanol, and deionized water in sequence for 10 min each. Ag NP dispersion (Nanopaste, NPS-J, Harima Chemicals Group, Inc., Japan) was dried, and then redissolved in anhydrous CF (Sigma Aldrich, ≥99%) at a concentration of 50 mg mL^−1^. Then, 5 wt% of 4Bx was added to the Ag NP solution in CF. To form the Ag NP S/D electrodes, a mixture solution of Ag NPs and 4Bx was spin-coated onto the PEN substrate at 1000 rpm for 30 s inside a N_2_-filled glove box. The resulting Ag NP film was exposed to a UV lamp (wavelength: 254 nm, power: 1000 W cm^−2^) for 30 s through a shadow mask. After photocrosslinking, the uncrosslinked regions were removed by spin-coating a fresh CF onto the film at 4000 rpm. As a result, the photocrosslinked regions of the Ag NP films were patterned directly on the PEN substrate and then thermally sintered in a vacuum oven at 150 °C for 8 h. The thickness of the sintered Ag NP electrode was around 72 nm. The channel length (*L*) and width (*W*) of the S/D electrodes were 100 and 800 µm, respectively. The photopatterning of all the subsequent components was performed in the same manner. In the case of the semiconducting polymer solutions, complete crosslinking could be successfully achieved by the addition of only 1 wt% of 4Bx. A 5 mg mL^−1^ blend solution of the *p*-type P(DPP2DT-TVT) polymer and 4Bx (1 wt%) was spin-coated onto the PEN substrate with the photopatterned Ag NP S/D electrodes. After the photopatterning of the *p*-type polymer film, the *n*-type P(NDI3OT-Se2) polymer film was formed by spin-coating a 5 mg mL^−1^ blend solution of *n-*type P(NDI3OT-Se2) and 4Bx (1 wt%). After the photopatterning of the *n*-type polymer film, both the photopatterned semiconducting polymer films were stored inside a glove box for 4 h to remove residual solvent. A 70 mg mL^−1^ mixed solution (PMMA (Sigma Aldrich, 120 kDa):4Bx = 95:5 wt%) in *n*-butyl acetate (Sigma Aldrich) was spin-coated to form the gate dielectric layer. After the photopatterning process of this layer, it was dried at 80 °C for 6 h in a vacuum oven. The thickness of the PMMA layer was around 482 nm. Finally, Ag NP gate electrodes were formed by the same method that was applied to form the Ag NP S/D electrodes.

## Supplementary information

Supplementary Information

## Data Availability

All data generated or analyzed during this study are included in this published article (and its Supplementary Information files).

## References

[1] Gelinck GH (2004). Flexible active-matrix displays and shift registers based on solution-processed organic transistors. Nat. Mater..

[2] Oh JY (2016). Intrinsically stretchable and healable semiconducting polymer for organic transistors. Nature.

[3] Yan H (2009). A high-mobility electron-transporting polymer for printed transistors. Nature.

[4] Sirringhaus H (2014). 25th anniversary article: organic field-effect transistors: the path beyond amorphous silicon. Adv. Mater..

[5] Kettner M (2019). Solution-processed organic transistors with excellent electrical stability under ambient conditions. Adv. Electron. Mater..

[6] Wang S (2018). Skin electronics from scalable fabrication of an intrinsically stretchable transistor array. Nature.

[7] Yao Y, Zhang L, Leydecker T, Samorì P (2018). Direct photolithography on molecular crystals for high performance organic optoelectronic devices. J. Am. Chem. Soc..

[8] Rogers JA, Someya T, Huang Y (2010). Materials and mechanics for stretchable electronics. Science.

[9] Xu X (2016). Electron mobility exceeding 10 cm^2^ V^−1^ s^−1^ and band‐like charge transport in solution-processed n-channel organic thin‐film transistors. Adv. Mater..

[10] Zhang G (2019). Fused heptacyclic-based acceptor-donor-acceptor small molecules: n-substitution toward high-performance solution-processable field-effect transistors. Chem. Mater..

[11] Paterson AF (2016). Small molecule/polymer blend organic transistors with hole mobility exceeding 13 cm^2^ V^−1^ s^−1^. Adv. Mater..

[12] Thomas TH (2019). Short contacts between chains enhancing luminescence quantum yields and carrier mobilities in conjugated copolymers. Nat. Commun..

[13] Nikolka M (2019). High-mobility, trap-free charge transport in conjugated polymer diodes. Nat. Commun..

[14] Fahlman M (2019). Interfaces in organic electronics. Nat. Rev. Mater..

[15] Khodagholy D (2013). High transconductance organic electrochemical transistors. Nat. Commun..

[16] Lee EK (2017). Chemically robust ambipolar organic transistor array directly patterned by photolithography. Adv. Mater..

[17] Hu X (2017). Directly photopatternable polythiophene as dual-tone photoresist. Macromolecules.

[18] Jacobs IE (2017). Direct-write optical patterning of P3HT films beyond the diffraction limit. Adv. Mater..

[19] DeFranco JA, Schmidt BS, Lipson M, Malliaras GG (2006). Photolithographic patterning of organic electronic materials. Org. Electron..

[20] Baeg K-J, Facchetti A, Noh Y-Y (2012). Effects of gate dielectrics and their solvents on characteristics of solution-processed n-channel polymer field-effect transistors. J. Mater. Chem..

[21] Minemawari H (2011). Inkjet printing of single-crystal films. Nature.

[22] Singh M, Haverinen HM, Dhagat P, Jabbour GE (2010). Inkjet printing-process and its applications. Adv. Mater..

[23] Guo LJ (2007). Nanoimprint lithography: methods and material requirements. Adv. Mater..

[24] Tsiminis G (2013). Nanoimprinted organic semiconductor laser pumped by a light‐emitting diode. Adv. Mater..

[25] Hermerschmidt F, Choulis SA, List-Kratochvil EJW (2019). Implementing inkjet-printed transparent conductive electrodes in solution-processed organic electronics. Adv. Mater. Technol..

[26] Sirringhaus H (2000). High-resolution inkjet printing of all-polymer transistor circuits. Science.

[27] Kahle FJ, Saller C, Köhler A, Strohriegl P (2017). Crosslinked semiconductor polymers for photovoltaic applications. Adv. Energy Mater..

[28] Rumer JW, McCulloch I (2015). Organic photovoltaics: crosslinking for optimal morphology and stability. Mater. Today.

[29] Reichmanis E, Thompson LF (1989). Polymer materials for microlithography. Chem. Rev..

[30] Keivanidis PE, Khong S-H, Ho PKH, Greenham NC, Friend RH (2009). All-solution based device engineering of multilayer polymeric photodiodes: minimizing dark current. Appl. Phys. Lett..

[31] Tao C (2013). Controlling hierarchy in solution‐processed polymer solar cells based on crosslinked P3HT. Adv. Energy Mater..

[32] Rumer JW (2015). Dual function additives: a small molecule crosslinker for enhanced efficiency and stability in organic solar cells. Adv. Energy Mater..

[33] Khong S-H (2007). General photo-patterning of polyelectrolyte thin films via efficient ionic bis (fluorinated phenyl azide) photo-crosslinkers and their post‐deposition modification. Adv. Funct. Mater..

[34] Liu B (2012). High internal quantum efficiency in fullerene solar cells based on crosslinked polymer donor networks. Nat. Commun..

[35] Png R-Q (2010). High-performance polymer semiconducting heterostructure devices by nitrene-mediated photocrosslinking of alkyl side chains. Nat. Mater..

[36] Cho N (2011). In-situ crosslinking and n-doping of semiconducting polymers and their application as efficient electron-transporting materials in inverted polymer solar cells. Adv. Energy Mater..

[37] Kim BJ, Miyamoto Y, Ma B, Frechet JMJ (2009). Photocrosslinkable polythiophenes for efficient, thermally stable, organic photovoltaics. Adv. Funct. Mater..

[38] Cai SX, Glenn DJ, Kanskar M, Wybourne MN, Keana JFW (1994). Development of highly efficient deep-UV and electron beam mediated cross-linkers: synthesis and photolysis of bis (perfluorophenyl) azides. Chem. Mater..

[39] Bräse S, Gil C, Knepper K, Zimmermann V (2005). Organic azides: an exploding diversity of a unique class of compounds. Angew. Chem. Int. Ed..

[40] Rodriquez D (2017). Comparison of methods for determining the mechanical properties of semiconducting polymer films for stretchable electronics. ACS Appl. Mater. Interfaces.

[41] Kim J-H (2013). Tensile testing of ultra-thin films on water surface. Nat. Commun..

[42] Zaumseil J, Sirringhaus H (2007). Electron and ambipolar transport in organic field-effect transistors. Chem. Rev..

[43] Kang MS, Frisbie CD (2013). A pedagogical perspective on ambipolar FETs. ChemPhysChem.

[44] Choi HH, Cho K, Frisbie CD, Sirringhaus H, Podzorov V (2017). Critical assessment of charge mobility extraction in fets. Nat. Mater..

[45] Noriega R (2013). A general relationship between disorder, aggregation and charge transport in conjugated polymers. Nat. Mater..

[46] Stucchi E, Dell’Erba G, Colpani P, Kim YH, Caironi M (2018). Low-voltage, printed, all-polymer integrated circuits employing a low-leakage and high-yield polymer dielectric. Adv. Electron. Mater..

[47] Kwon J (2019). Three-dimensional monolithic integration in flexible printed organic transistors. Nat. Commun..

