# Supplementary material for: Universal three-dimensional crosslinker for all-photopatterned electronics
Source: Nat Commun. 2020 Mar 23;11:1520. doi: 10.1038/s41467-020-15181-4 (PMC7089981; doi:10.1038/s41467-020-15181-4)
Supplement: Supplementary file 1 — Supplementary Information [file 41467_2020_15181_MOESM1_ESM.docx]

**Universal Three-Dimensional Crosslinker for All- Photopatterned Electronics**

**Kim, M. J. et al**

**Supplementary Information**

**Universal Three-Dimensional Crosslinker for All-Photopatterned Electronics**

Min Je Kim,^1,†^ Myeongjae Lee,^2,†^ Honggi Min,^3^ Seunghan Kim,^4^ Jeehye Yang,^4^ Hyukmin Kweon,^5^ Wooseop Lee,^3^ Do Hwan Kim,^5^ Jong-Ho Choi,^2^ Du Yeol Ryu,^3^ Moon Sung Kang,^4,^* BongSoo Kim,^6,^* Jeong Ho Cho^3,^*

^1^SKKU Advanced Institute of Nanotechnology (SAINT), Sungkyunkwan University (SKKU), Suwon 16419, Republic of Korea.

^2^Department of Chemistry, Korea University, Seoul 02841, Republic of Korea.

^3^Department of Chemical and Biomolecular Engineering, Yonsei University, Seoul 03722, Republic of Korea.

^4^Department of Chemical and Biomolecular Engineering, Sogang University, Seoul 04107, Republic of Korea.

^5^Department of Chemical Engineering, Hanyang University, Seoul 04763, Republic of Korea.

^6^Department of Chemistry, Ulsan National Institute of Science and Technology (UNIST), Ulsan 44919, Republic of Korea.

^†^These authors contributed equally to this work.

*Corresponding authors.

E-mail: M. S. Kang ([kangms@sogang.ac.kr](mailto:kangms@sogang.ac.kr)), B. Kim ([bongsoo@unist.ac.kr](mailto:bongsoo@unist.ac.kr)), and J. H. Cho ([jhcho94@yonsei.ac.kr](mailto:jhcho94@yonsei.ac.kr))

**Supplementary Methods**

**General information**

*Synthetic Materials:* Tris(dibenzylideneacetone) dipalladium(0) (Pd_2_(dba_3_)), tetrakis(triphenylphosphine) palladium(0) (Pd(PPh_3_)_4_), anhydrous toluene, anhydrous chlorobenzene, and anhydrous ethylene glycol were purchased from Sigma Aldrich. *N,N*-dimethylformamide (DMF) was purchased from ACROS. Toluene, chlorobenzene, and DMF, solvents for polymerization, were separately degassed by three freeze-pump-thaw cycles and added to reaction mixtures. Diethylammonium diethyldithiocarbamate, triphenylphosphine, 2-bromothiopehene, and pentaerythriol were purchased from Alfa Aesar. 4-azido-2,3,5,6-tetrafluorobenzoic acid and triethyl amine were purchased from TCI. Thionyl chloride and rest of other solvent were purchased from Daejung and used as received.

4,9-Dibromoisochromeno[6,5,4-*def*]isochromene-1,3,6,8-tetraone (NDA-Br2) was purchased from SunaTech. Inc. (China). 3,6-bis(5-bromothiophen-2-yl)-2,5-bis(2-decyltetradecyl)pyrrolo[3,4-*c*]pyrrole-1,4(2*H*,5*H*)-dione (DPP2DT-Br2), 2,5-bis(trimethylstannyl)selenophene, 5,5'-bis(trimethylstannyl)-2,2'-biselenophene, PTB7-Th, P(DPP2DT-F2T2), P(DPP2DT-T2), P(DPP2DT-TVT), P(DPP2DT-F2T2) P(NDI2OD-F2T2), and P(NDI2OD-Se2) were synthesized by following literature methods.^1-4^

**Characterization of Synthesized Molecules and Polymers**

To identify the molecular structures of all the synthesized products, ^1^H-NMR, ^13^C-NMR, and ^19^F-NMR were measured by Bruker Avance III 300, 75, and 282 MHz, respectively, with deuterated chloroform (CDCl_3_) as solvent, which was purchased from Cambridge Isotope Laboratories. Mass spectra of 2,2-bis(((4-azido-2,3,5,6-tetrafluorobenzoyl)oxy)methyl)propane-1,3-diyl bis(4-azido-2,3,5,6-tetrafluorobenzo-ate) (**4Bx**) was obtained the gas-phase fast atom bombardment (FAB) ionization technique using Xe beam at the Korea Basic Science Institute (KBSI), Daegu Branch, Republic of Korea. Molecular weight and polydispersity index (PDI) of synthesized polymers were determined by an Agilent GPC system (GPC 1200 system) run at 80°C. *o*-Dichlorobenzene was used as the eluent, and polystyrene standards were used for molecular weight calibration.

**Supplementary Figure 1** Synthetic routes to 2Bx and 4Bx.

**Synthesis of ethane-1,2-diyl bis(4-azido-2,3,5,6-tetrafluorobenzoate), 2Bx**

A mixture of 4-azido-2,3,5,6-tetrafluorobenzoic acid (200 mg, 0.8507 mmol) and SOCl_2_ (1 M in dichloromethane) (0.12 mL, 1.701 mmol) in anhydrous dichloromethane (2.4 mL) was refluxed at 70°C in an oil bath. After 12 h, the reaction mixture was cooled down to rt and then the organic solvents were removed by distllation at a reduced pressure. Next, the crude acyl chloride compound were dissolved in anhydrous dichloromethane (2.4 mL) and transferred to a mixture of ehtylene glycol (22 mg, 0.3545 mmol) and triethylamine (86.08 mg, 0.8507 mmol) in anhydrous dichloromethane (10 mL). After this reaction mixture was stirred for 4 h at rt, it was quenched with water (10 mL). The aqueous phase was extracted with dichloromethane (10 mL × 3). The organic phases were combined and washed with brine (25 mL) and dried over MgSO_4_ and filtered, and the filtatrate was dried by a rotary evaoporator. The resulting crude product was purified by silica gel column chromatography using an eluent of ethyl acetate/*n*-hexane (1/5 to 1/3), which affored the **2Bx** as a white solid (105.8 mg, 60%). ^1^H-NMR (300 MHz, CDCl_3_): *δ* = 4.685 (s, 4H). ^19^F-NMR (282 MHz, CDCl_3_): *δ* = -138.128-138.185 (m), -150.634-150.765 (m). The spectral data are well matched with the literature.^5^


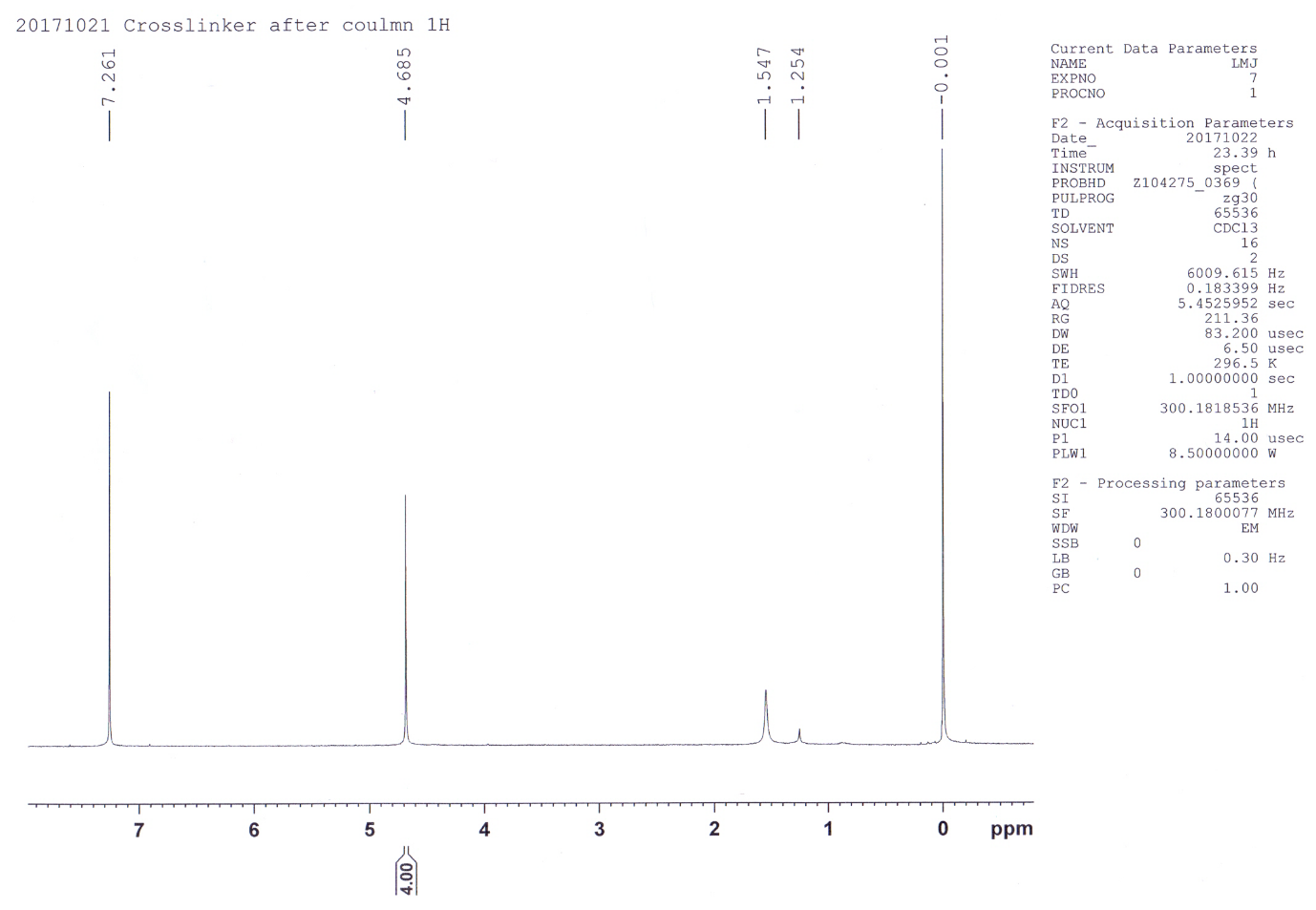


**Supplementary Figure 2** ^1^H-NMR spectrum of 2Bx.


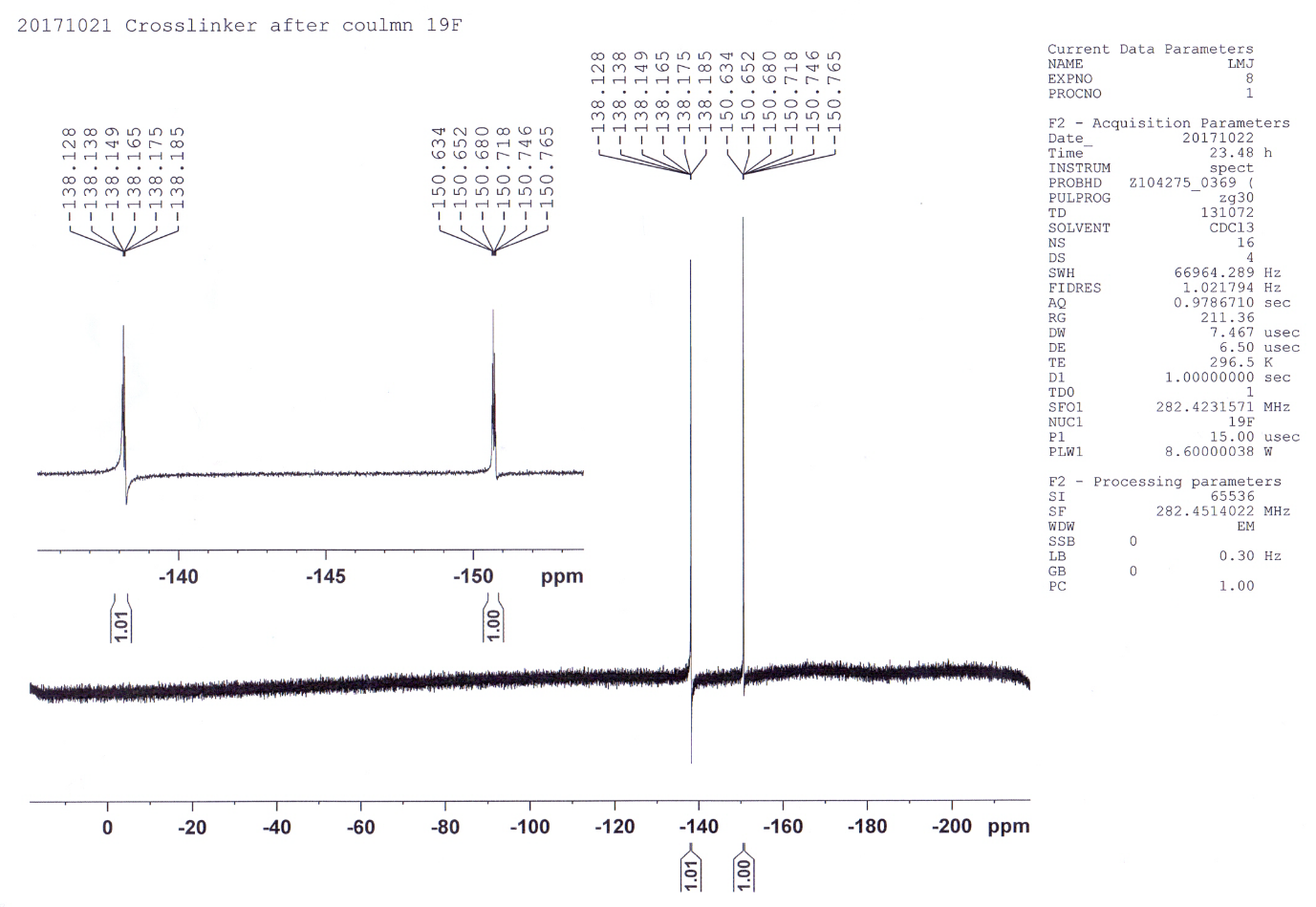


**Supplementary Figure 3** ^19^F-NMR spectrum of 2Bx.

**Synthesis of 2,2-bis(((4-azido-2,3,5,6-tetrafluorobenzoyl)oxy)methyl)propane-1,3-diyl bis(4-azido-2,3,5,6-tetrafluorobenzoate), 4Bx**

A mixture of 4-azido-2,3,5,6-tetrafluorobenzoic acid (1000 mg, 4.2536 mmol) and SOCl_2_ (0.62 mL, 8.5072 mmol) in anhydrous dichloromethane (25 mL) was refluxed at 70°C in an oil-bath. After 12 h, the reaction mixture was cooled down to rt and the organic solvents were removed by distllation at a reduced pressure. Next, the crude acyl chloride compound were dissolved in anhydrous dichloromethane (18 mL) and transferred to a mixture of 2,2-bis(hydroxymethyl)propane-1,3-diol (120.65 mg, 0.8862 mmol) and triethylamine (430.42 mg, 4.2536 mmol) in anhydrous dichloromethane (8 mL). (※ 2,2-bis(hydroxymethyl)propane-1,3-diol was ground by mortar before use because of its poor solubility in dichloromethane.) After this reaction mixture was stirred for 28 h at rt, it was quenched with water (30 mL). The aqueous phase was extracted with dichloromethane (25 mL × 3). The organic phases were combined and washed with brine (80 mL) and dried over MgSO_4_ and filtered, and the filtatrate was dried by a rotary evaoporator. The resulting crude product was purified by silica gel column chromatography using an eluent of ethyl acetate/*n*-hexane (1/5 to 1/3), which affored the 4-Bx as a white solid (792.7mg, 89%). ^1^H-NMR (300 MHz, CDCl_3_) *δ*: 4.556 (s, 4H). ^19^F-NMR (282 MHz, CDCl_3_) *δ*: -137.920-138.051 (m), -150.188-150.319 (m). HR-FAB mass calcd for C_33_H_8_F_16_N_12_O_8_ [M + H]^+^ : 1005.0411, found: *m/z* 1005.0408.


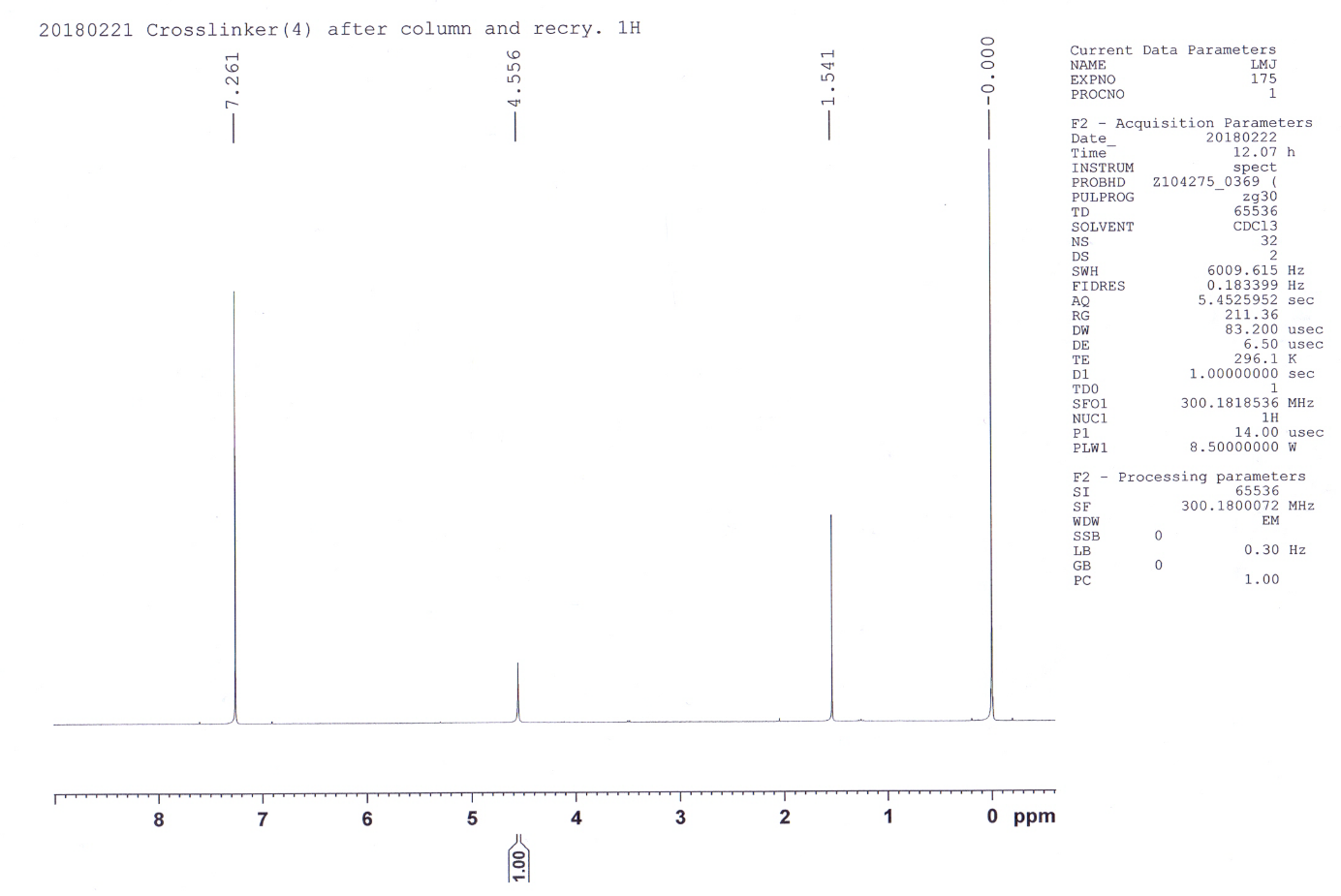


**Supplementary Figure 4** ^1^H-NMR spectrum of 4Bx.


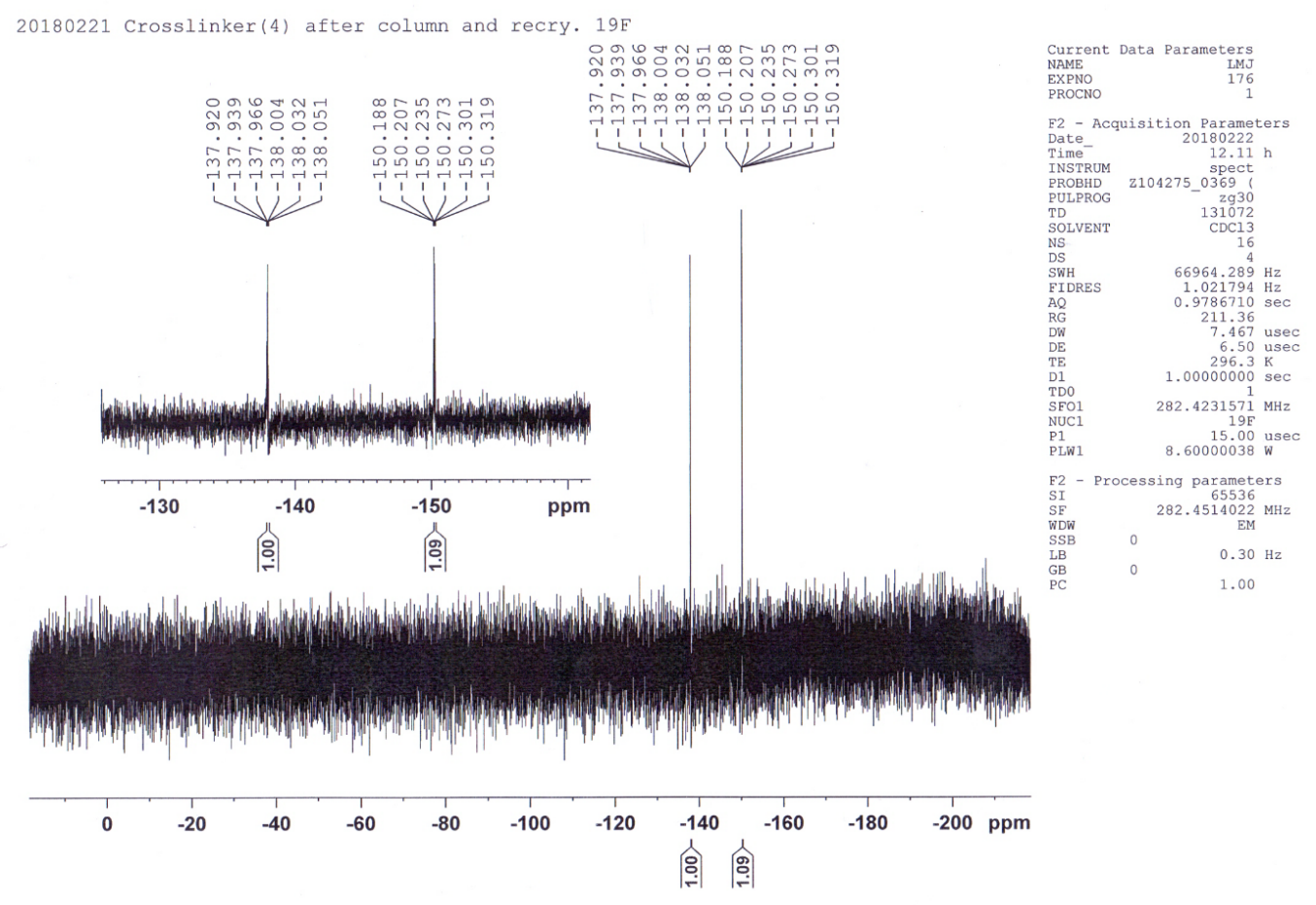


**Supplementary Figure 5** ^19^F-NMR spectrum of 4Bx.


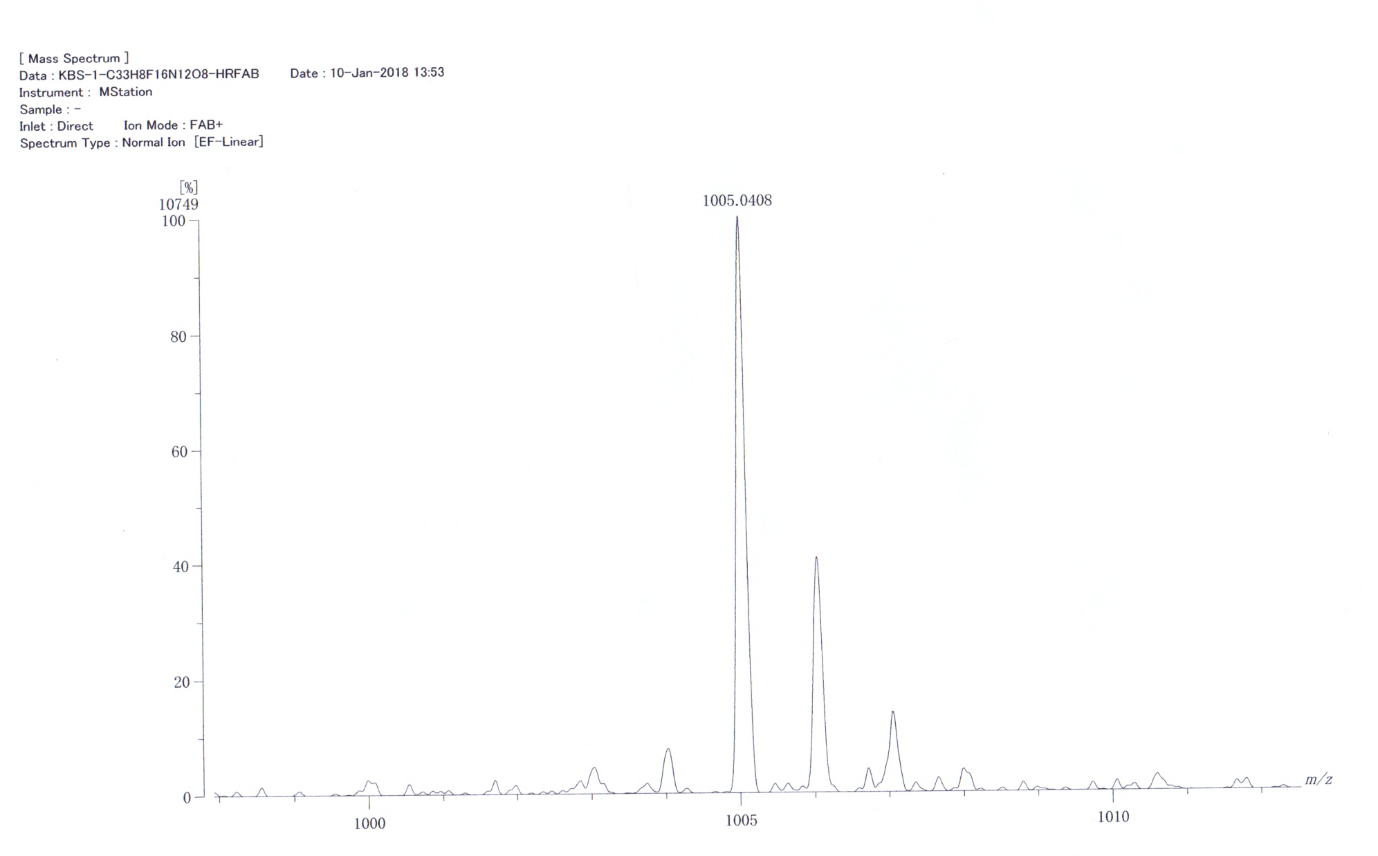


**Supplementary Figure 6** HR-FAB spectrum of 4Bx.

**Supplementary Figure 7** Synthetic scheme of P(DPP2DT-TVT).

**Synthesis of poly((E)-2,5-bis(2-decyltetradecyl)-3-(thiophen-2-yl)-6-(5'-(2-(thiophen-2-yl)vinyl)-[2,2'-bithiophen]-5-yl)pyrrolo[3,4-c]pyrrole-1,4(2H,5H)-dione), P(DPP2DT-TVT)**

DPP2DT-Br2 (300 mg, 0.2652 mmol), (*E*)-1,2-bis(5-(trimethylstannyl)thiophen-2-yl)ethene (137.35 mg, 0.2652 mmol), tris(dibenzylideneacetone)dipalladium(0) (7.29 mg, 3 mol%) and triphenylphosphine (8.35 mg, 12 mol%) were dissolved in chlorobenzene (10 mL) and the solution was stirred at 60°C. The reaction solution was heated up to 100°C gradually at a rate of 1°C/min. After 80 min, the reaction temperature was heated up to 118°C. After 110 min, DMF (1 mL) was added to the solution. After 290 min, 2-bromothiophene (0.1 mL) was added to the reaction solution which then stirred for another 1 h. Next, the reaction solution was transferred to 100 mL RB flask using chloroform (10 mL) and diethylammonium diethyldithiocarbamate solution (1.0 M) in water (30 mL) was added to the flask and then was stirred 1 h at 50°C. After cooling to rt, organic layer was separated and washed three times with water (30 mL) followed by brine (30 mL). The organic layer was dried by a rotary evaporator and then the crude polymer was precipitated in methanol (300 mL). The precipitated polymer was filtered through a thimble tube and then purified by Soxhlet extraction with methanol, acetone, *n*-hexane, cyclohexane, dichloromethane, and chloroform. The cyclohexane, dichloromethane, and chloroform fractions were precipitated in methanol (300 mL). The resulting precipitates were filtered and dried under vacuum to yield P(DPP2DT-TVT) batches: cyclohexane: 33.3 mg (11%), dichloromethane: 221.7 mg (72%), chloroform: 46.7 mg (15%). ^1^H-NMR (300 MHz, CDCl_3_) *δ*: 9.52-8.35 (4H), 7.18-5.85 (4H), 2.04-0.48 (98H). Cyclohexane batch GPC (*o*-dichlorobenzene, 80°C) M_n_ = 3,700 Da, M_w_ = 3,900 Da, PDI = 1.08, dichloromethane batch GPC (*o*-dichlorobenzene, 80°C) M_n_ = 11,000 Da, M_w_ = 38,000 Da, PDI = 3.5, and chloroform batch GPC (*o*-dichlorobenzene, 80°C) M_n_ = 24,000 Da, M_w_ = 110,000 Da, PDI = 4.7.


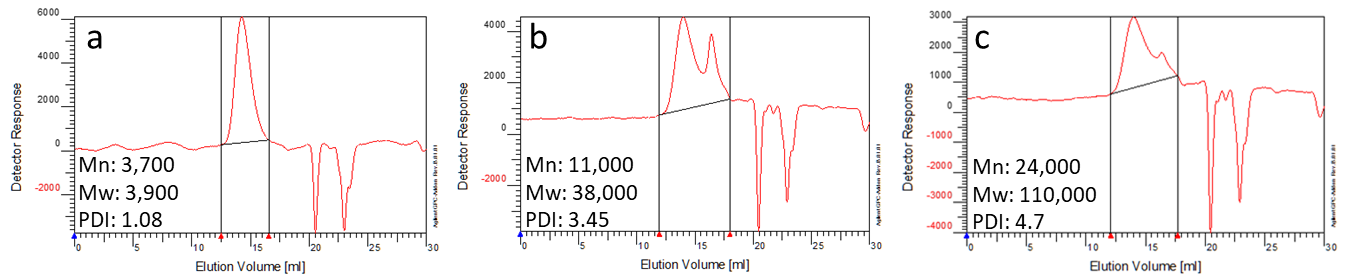


**Supplementary Figure 8** **Gel permeation chromatograms of P(DPP2DT-TVT) polymers** **a**, cyclohexane batch, (**b**) dichloromethane batch, and (**c**) chloroform batch.

**Supplementary Figure 9** Synthetic scheme of P(NDI3OT-Se) and P(NDI3OT-Se2).

**Synthesis of 9-(iodomethyl)nonadecane, 1**

To a stirred solution of 2-octyldodecan-1-ol (2OD-OH) (10 g, 33.495 mmol), imidazole (2.736 g, 40.194 mmol), and triphenylphosphine (10.543 g, 40.194 mmol) in dichloromethane (63 mL) was slowly added iodine (10.2017 g, 40.194 mmol) at 0°C and then the reaction mixture was stirred at rt for 14 h. The reaction solution was quenched by sat. Na_2_S_2_O_3(aq)_ solution (30 mL). The organic layer was washed by brine (50 mL) and by water (50 mL) three times, and then dried over anhydrous MgSO_4_. After the organic solvents were evaporated, crude product was purified by silica plug with *n*-hexane as an eluent. Finally, the resulting fraction was concentrated under reduced pressure using a rotary evaporator and further dried in a high vacuum chamber to afford the pure product as a colorless oil (13.62 g, 99.5%). ^1^H-NMR (300 MHz, CDCl_3_): *δ* = 3.29 (d, 2H), 1.40-1.19 (m, 32H), 1.17-1.09 (br, 1H), 0.92-0.83(t, 6H). The ^1^H-NMR spectra are well matched with reported ^1^H-NMR data in the literature.^6^

**Synthesis of 3-octyltridecanenitrile, 2**

To a stirred solution of **1** (10 g, 24.48 mmol) in ethanol (100 mL) and H_2_O (10 mL) was added cyanopotassium (4.78 g, 73.44 mmol). The reaction solution was refluxed at 90°C for 13 h. The reaction was cooled down to rt. The organic layer was extracted with *n*-hexane (80 mL) four times, and then dried over anhydrous MgSO_4_. The resulting organic layer was concentrated under reduced pressure using a rotary evaporator and further dried in a high vacuum chamber to afford crude product, as a colorless oil (7.3 g, 96.8%). The crude product was pure enough for the next reaction. ^1^H-NMR (300 MHz, CDCl_3_): *δ* = 2.32-2.28 (d, 2H), 1.72-1.58 (m, 1H), 1.40-1.18 (m, 32H), 0.91-0.84 (t, 6H). ^13^C-NMR (75 MHz, CDCl_3_): *δ =* 119.01, 35.11, 33.52, 31.91, 31.87, 29.67, 29.61, 29.55, 29.51, 29.34, 29.27, 26.60, 22.70, 22.67, 21.72, 14.12.


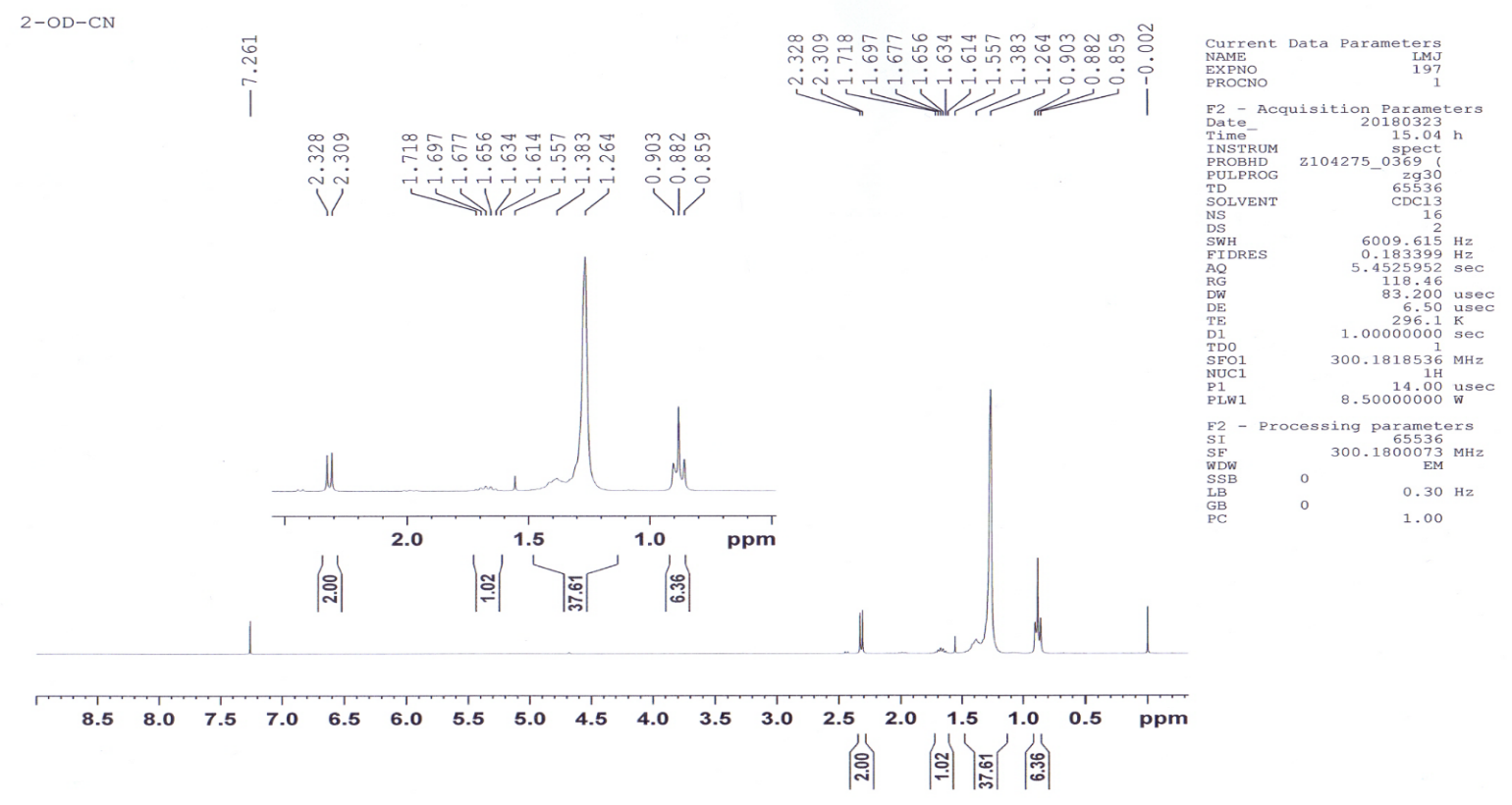


**Supplementary Figure 10** ^1^H-NMR of 2.


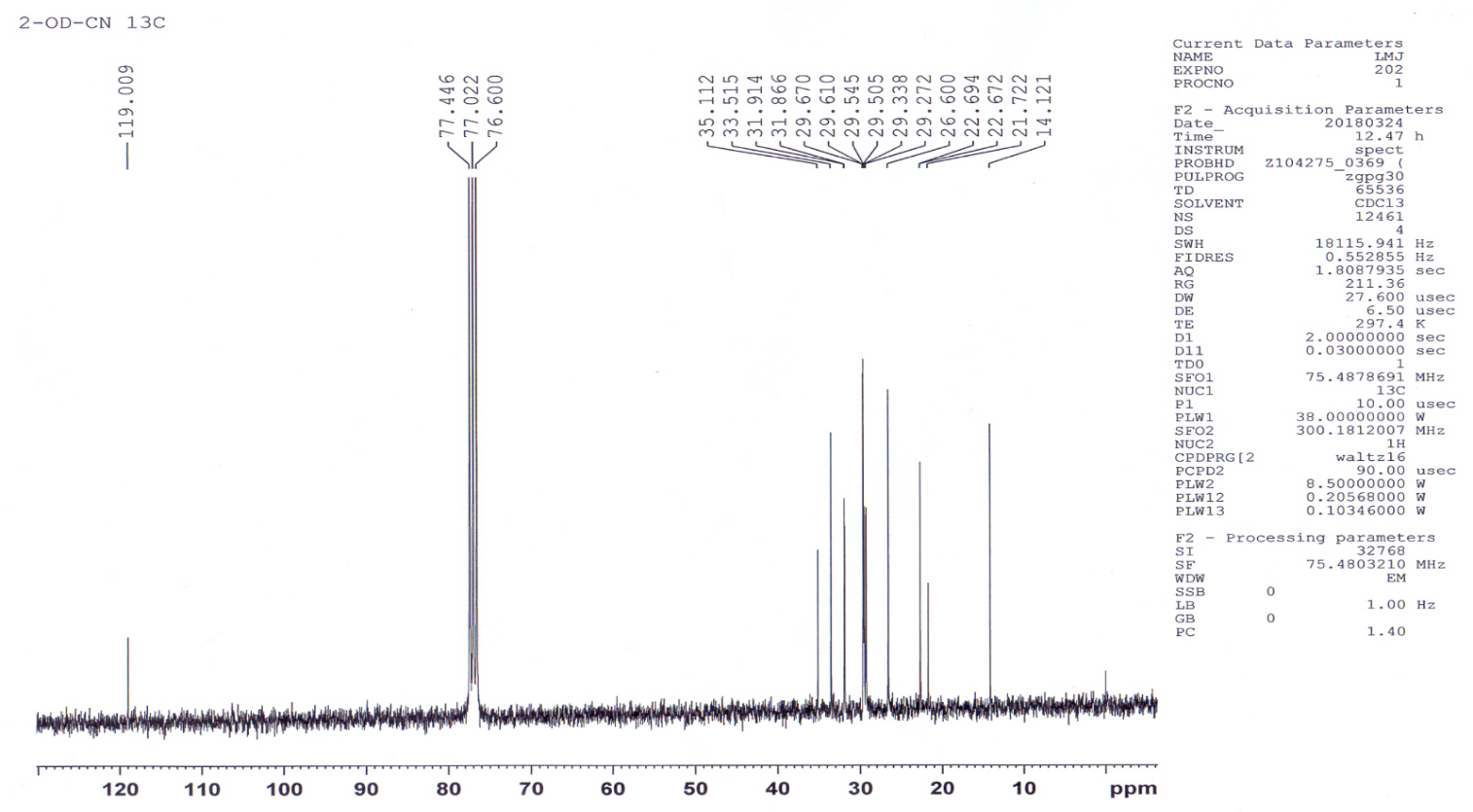


**Supplementary Figure 11** ^13^C-NMR of 2.

**Synthesis of 3-octyltridecan-1-amine, 3**

To a stirred solution of **2** (7 g, 22.76 mmol) in THF (46 mL) was slowly added LiAlH_4_ (2.0 M in THF) (22.8 mL, 45.52 mmol) at 0°C. After 20 min, the reaction solution was warmed up to rt and stirred for 1 h, and then refluxed at 70°C for 13 h. The reaction mixture was cooled down to 0°C and H_2_O (15 mL) was added until no further gas evolution was observed. The reaction mixture was filtered through Celite, and the resulting solution was extracted with *n*-hexane (50 mL) three times and then the organic layer was dried over anhydrous MgSO_4_. The resulting organic layer was concentrated under reduced pressure using a rotary evaporator and further dried in a high vacuum chamber to afford product as a colorless oil (6.6 g, 93%). The product was used for the next step without further purification. ^1^H-NMR (300 MHz, CDCl_3_): *δ* = 2.71 (t, 2H), 1.41-1.19 (m, 32H), 0.91-0.80 (m, 6H). ^13^C-NMR (75 MHz, CDCl_3_): *δ =* 40.10, 38.22, 35.30, 33.76, 31.93, 30.12, 29.71, 29.67, 29.37, 26.62, 22.70, 14.13.


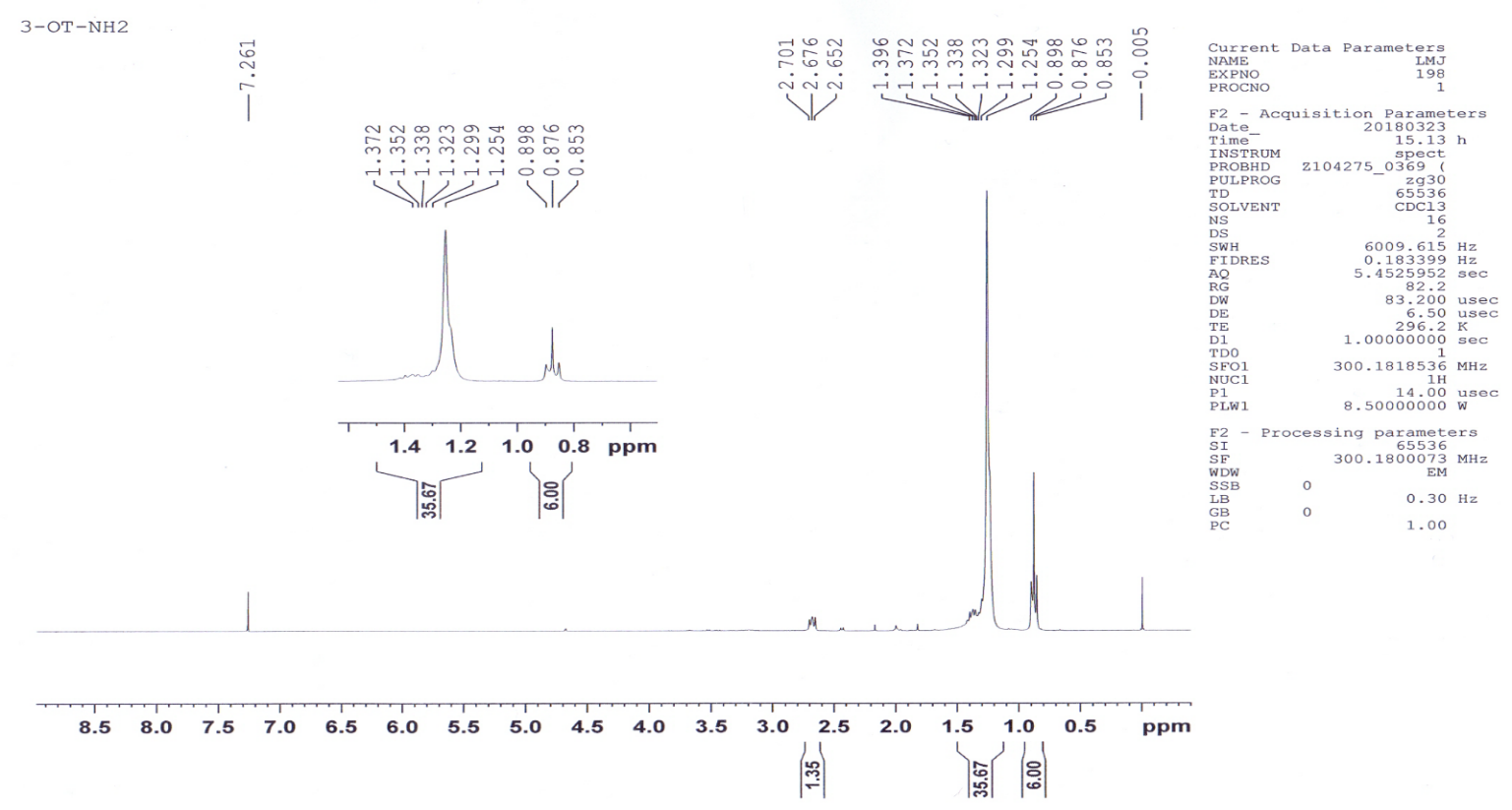


**Supplementary Figure 12** ^1^H-NMR of 3.


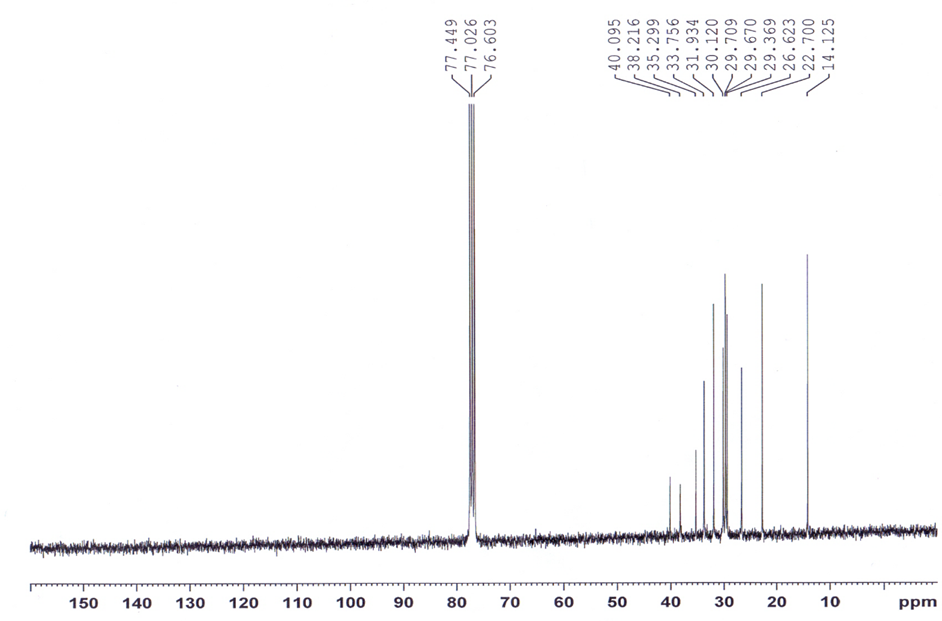


**Supplementary Figure 13** ^13^C-NMR of 3.

**Synthesis of 4,9-dibromo-2,7-bis(3-octyltridecyl)benzo[lmn][3,8]phenanthroline-1,3,6,8(2H,7H)-tetraone, NDI3OT-Br2**

To a stirred solution of NDA-Br2 (2.0 g, 4.70 mmol) in acetic acid (60 mL) was added dropwise **3** (5.12 g, 16.43 mmol). The reaction solution was refluxed at 120°C for 16 h. The reaction temperature was cooled down to rt. Water (40 mL) was added to the reaction solution, which was then extracted with ethyl acetate (40 mL) three times, washed by brine (50 mL) and by water (50 mL). The resulting organic phase was dried over anhydrous MgSO_4_ and the organic solvent was evaporated using a rotary evaporator. The crude product was precipitated in ethanol and stored in 2°C fridge. After 12 h, the precipitate was filtered. The collected red color crude product was purified by column chromatography silica with ethyl acetate:*n*-hexane (1:3). Finally, the collected product was recrystallized in DCM/ethanol and formed crystals were filtered, dried vacuum to afford the pure product as a yellow solid (2.75 g, 58%). ^1^H-NMR (300 MHz, CDCl_3_): *δ* = 8.98 (s, 2H), 4.25-4.10 (m, 4H), 1.71-1.60 (m, 4H), 1.470 (br, 2H), 1.35-1.24 (m, 64H), 0.95-0.80 (t, 12 H). ^13^C-NMR (75 MHz, CDCl_3_): *δ =* 160.68, 160.64, 139.02, 128.28, 127.71, 125.37, 124.13, 40.01, 36.05, 33.54, 31.94, 31.70, 30.07, 29.70, 29.68, 29.39, 26.61, 22.72, 14.15.


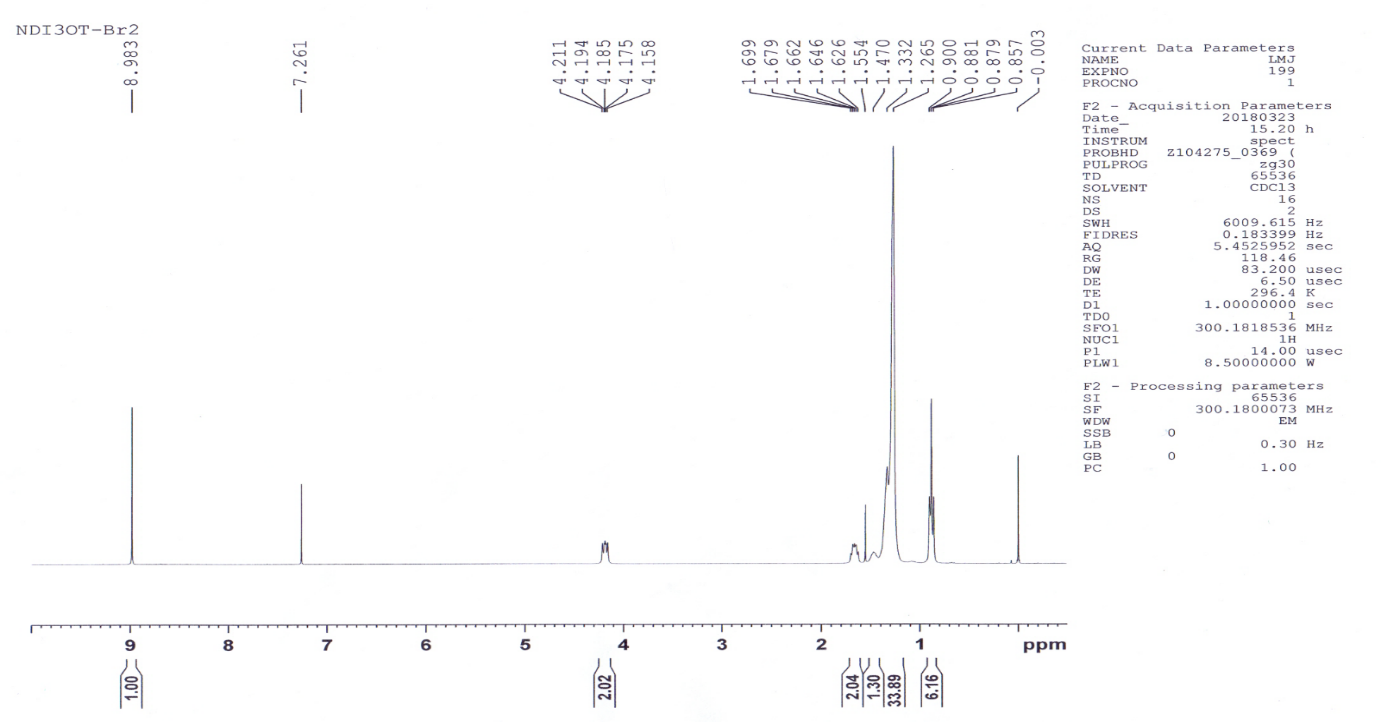


**Supplementary Figure 14** ^1^H-NMR of NDI3OT-Br2.


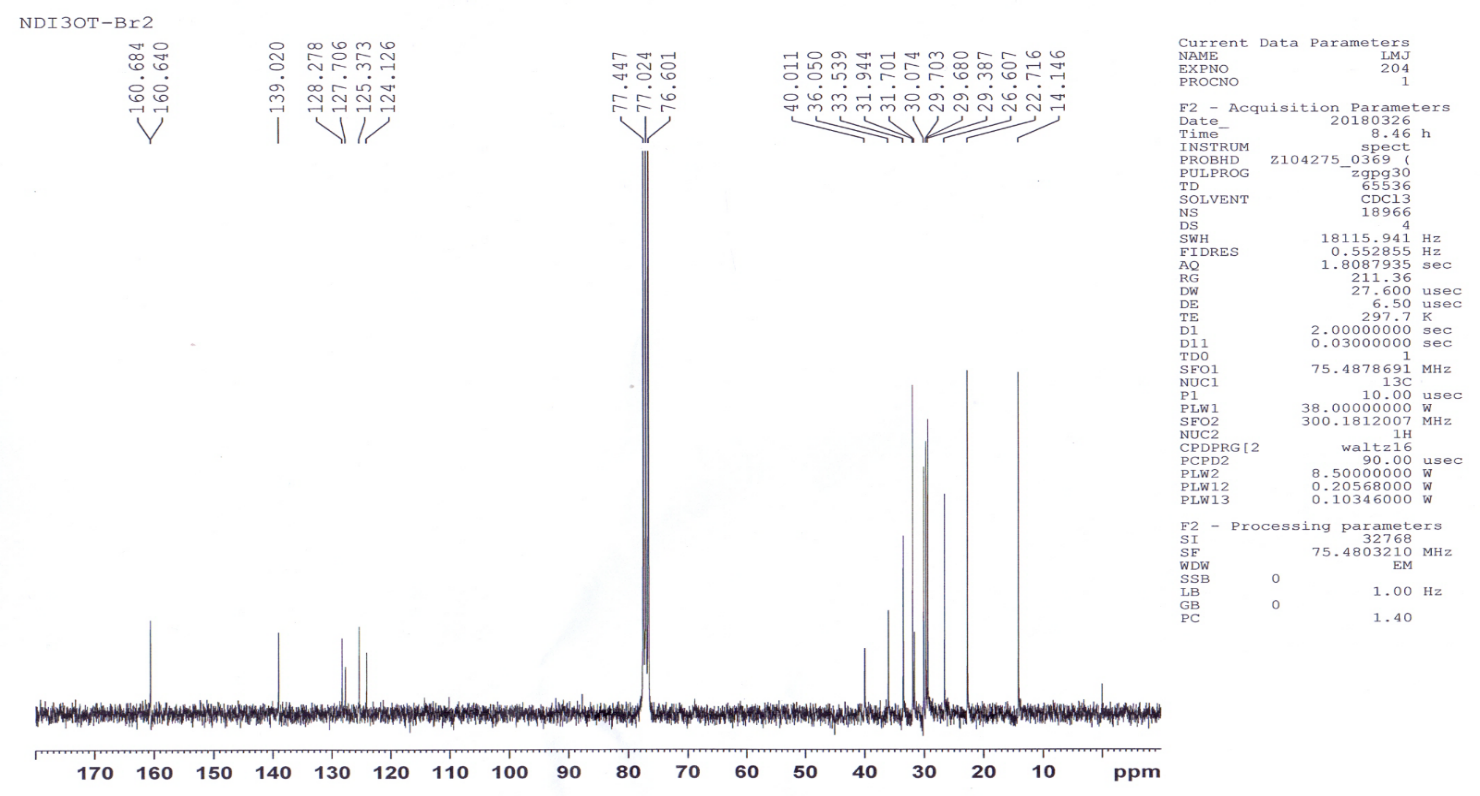


**Supplementary Figure 15** ^13^C-NMR of NDI3OT-Br2.

**Synthesis of poly(2,7-bis(3-octyltridecyl)-4-(selenophen-2-yl)benzo[lmn][3,8]phenanthroline-1,3,6,8(2H,7H)-tetraone), P(NDI3OT-Se)**

4,9-dibromo-2,7-bis(3-octyltridecyl)benzo[*lmn*]-[3,8]phenanthroline-1,3,6,8(2*H*,7*H*)-tetraone (NDI3OT-Br2) (0.150 g, 0.148 mmol), 2,5-bis(trimethylstannyl)selenophene (0.067 g, 0.148 mmol), tris(dibenzylideneacetene) dipalladium(0) (0.004 g, 3 mol%), and triphenylarsine (0.005 mg, 12 mol%) were dissolved in degassed chlorobenzene (2.3 mL). The solution was heated with stirring from rt to 60°C rapidly and then to 90°C gradually at a rate of 1°C/2 min. After 25 min polymerization, 2-bromothiophene (0.05 mL) was added to the solution and stirred for 2 h. The reaction mixture was cooled down to rt and transferred to 50 mL R.B. flask using chloroform (9 mL). Then, diethylammonium diethyldithiocarbamate (8 mg) in H_2_O (12 mL) was added to the R.B. flask and stirred at 50°C for 26 h. The solution was extracted with chloroform (12 mL) three times. The organic phase was washed with brine (25 mL), and with water (25 mL). The organic solvent was removed under reduced pressure. The dried crude polymer was re-dissolved using chloroform (6 mL) and then precipitated in methanol (200 mL). The collected polymer was further purified by Soxhlet extraction using methanol, acetone, *n*-hexane, cyclohexane, and dichloromethane. The dichloromethane fraction was precipitated in methanol (200 mL). The precipitated DCM batch was filtered and dried under a vacuum condition to yield blue-color polymer P(NDI3OT-Se) (0.122 g, 84.1%). ^1^H-NMR (300 MHz, CDCl_3_): δ = 9.05-8.95 (br, 2H), 7.75-7.60 (br, 1H), 4.40-3.85 (br, 4H), 1.81-1.55 (br, 4H), 1.44-0.93 (br, 80H), 0.92-0.76 (br, 12H). GPC (*o*-dichlorobenzene, 80°C): M_n_ = 39,000 Da, M_w_ = 82,000 Da, PDI = 2.1.


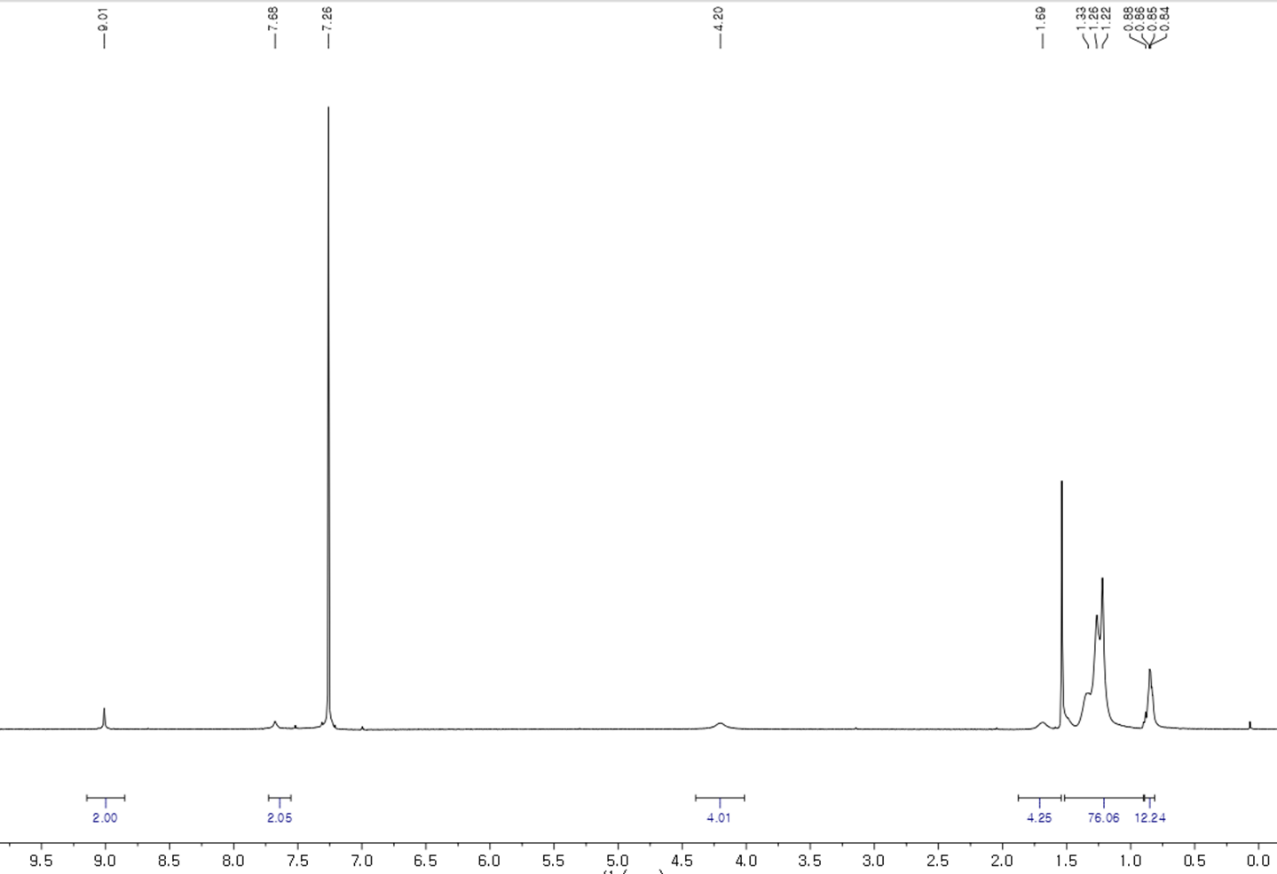


**Supplementary Figure 16** ^1^H-NMR of P(NDI3OT-Se).


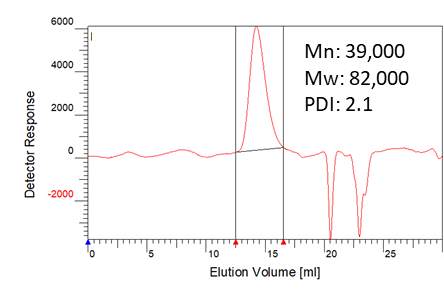


**Supplementary Figure 17** Gel permeation chromatogram of P(NDI3OT-Se).

**Synthesis of low molecular weight and high molecular weight poly(4-([2,2'-biselenophen]-5-yl)-2,7-bis(3-octyltridecyl)benzo[lmn][3,8]phenanthroline-1,3,6,8(2H,7H)-tetraone), 73 and 134 kD P(NDI3OT-Se2)**

NDI3OT-Br2 (0.150 g, 0.148 mmol), 2,5-bis(trimethyltin)-selenophene (0.087 g, 0.148 mmol), tris(dibenzylideneacetene)dipalladium(0) (0.004 g, 3 mol%), and triphenylarsine (0.005 mg, 12 mol%) were dissolved in degassed chlorobenzene (2.3 mL). The solution was heated with stirring from rt to 60 °C and then to 70°C gradually at a rate of 1°C/2 min. After 6 min polymerization, 2-bromothiophene (0.05 mL) was added to the solution and then stirred for 2 h. The reaction mixture was cooled down to rt and transferred to 50 mL R.B flask using chloroform (11 mL) and then diethylammonium diethyldithiocarbamate (8 mg) in H_2_O (12 mL) was added to the R.B. flask and stirred at 50°C for 26 h. The solution was extracted with chloroform (15 mL) three times. . The organic phase was washed with brine (30 mL) and with water (30 mL). The organic solvent was removed under reduced pressure. The dried crude polymer was re-dissolved using chloroform (8 mL) and then precipitated in methanol (200 mL). The collected polymer was further purified by Soxhlet extraction using methanol, acetone, *n*-hexane, cyclohexane, dichloromethane, and chloroform. The dichloromethnae and chloroform fractions were precipitated in methanol (200 mL). The precipitated dichloromethane and chloroform batchs were filtered and dried under a vacuum condition to yield deep blue-color polymer P(NDI3OT-Se2). Dichloromethane batch (74.2 mg, 45.1%) and chloroform batch (80 mg, 48.7%). ^1^H-NMR (300 MHz, CDCl_3_): *δ* = 9.00-8.55 (2H), 7.6-7.25 (4H), 2.04-0.44 (84H). GPC (*o*-dichlorobenzene, 80°C): dichloromethane batch M_n_ = 25,000 Da, M_w_ = 73,000 Da, PDI = 2.9, chloroform batch M_n_ = 44,000, M_w_ = 134,000 Da, PDI = 3.04.


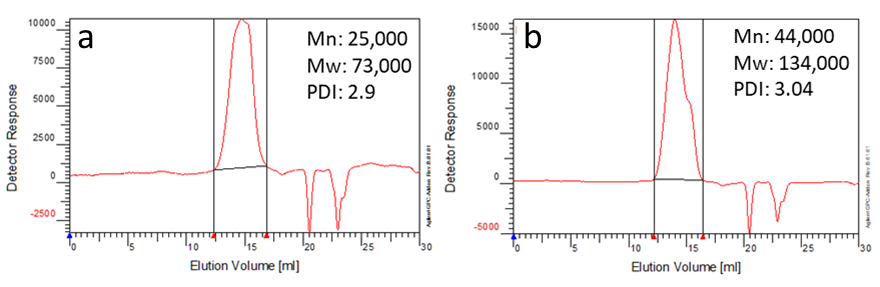


**Supplementary Figure 18 Gel permeation chromatogram of the P(NDI3OT-Se2)** **a**, dichlromethane batch and (**b**) chloroform batch.

**
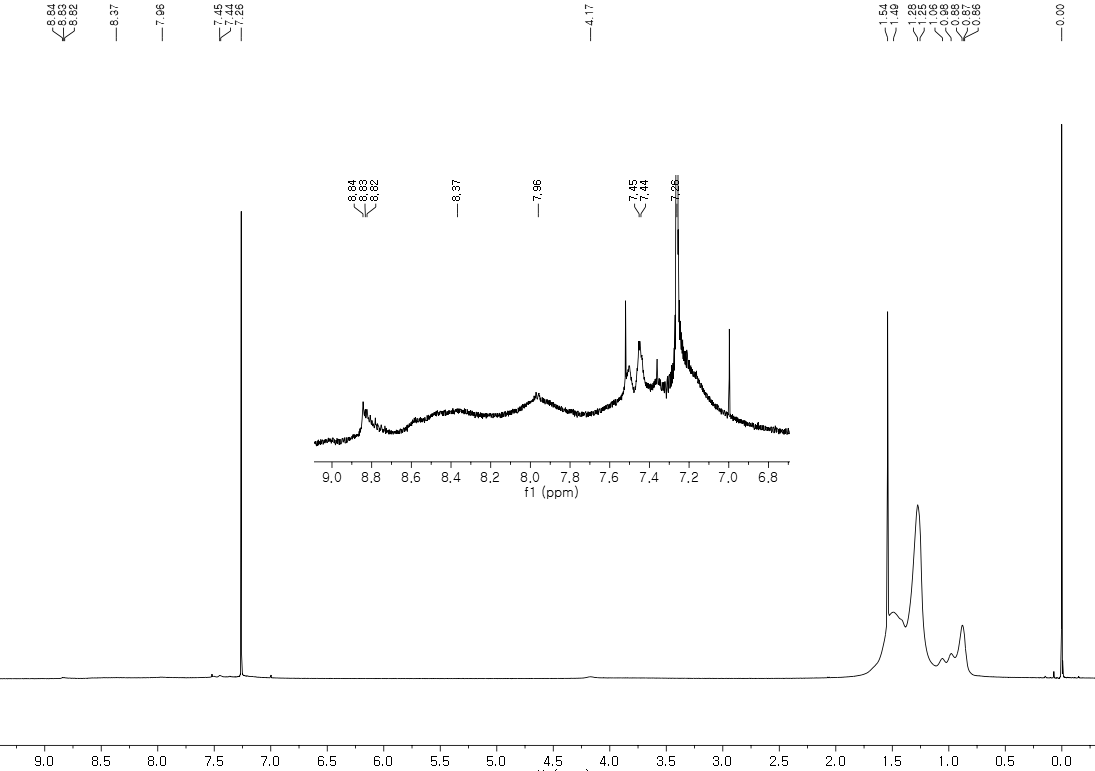
**

**Supplementary Figure 19** ^1^H-NMR of 134 kD P(NDI3OT-Se2).

**Synthesis of poly(4-([2,2'-biselenophen]-5-yl)-2,7-bis(3-octyltridecyl)benzo[lmn][3,8]phenan-throline-1,3,6,8(2H,7H)-tetraone), 477 kD P(NDI3OT-Se2)**

NDI3OT-Br2 (0.500 g, 0.4935 mmol), 2,5-bis(trimethyltin)-selenophene (0.289 g, 0.4935mmol), tris(dibenzylideneacetene) dipalladium(0) (0.013 g, 3 mol%), and triphenylarsine (0.018 g, 12 mol%) were dissolved in degassed chlorobenzene (7.42 mL). The solution was heated with stirring from rt to 60°C rapidly and then to 76°C gradually at a rate of 1°C/2 min. After 30 min polymerization, 2-bromothiophene (0.2 mL) was added to the solution and then stirred for 2 h. The reaction mixture was cooled down to rt and transferred to 250 mL R.B. flask using chloroform (30 mL) and then diethylammonium diethyldithiocarbamate (30 mg) in H_2_O (35 mL) was added to the R.B. flask and stirred at 50°C for 28 h. The solution was extracted with chloroform (45 mL) three times. The organic phase was washed with brine (40 mL) and with water (40 mL). The organic solvent was removed under reduced pressure. The dried crude polymer was re-dissolved using chloroform (15 mL) and then was precipitated in methanol (200 mL). The collected polymer was further purified by Soxhlet extraction using methanol, acetone, *n*-hexane, cyclohexane, dichloromethane, and chloroform. The chloroform fraction were precipitated in methanol. The precipitated chloroform batch was filtered and dried under a vacuum condition to yield deep blue-color polymer 477 kD P(NDI3OT-Se2) (0.518 g, 94.4%). ^1^H-NMR (300 MHz, CDCl_3_): *δ* = 9.52-8.35 (4H), 7.18-5.85 (4H), 2.04-0.48 (98H). GPC (*o*-dichlorobenzene, 80°C): chloroform batch M_n_ = 84,000 Da, M_w_ = 477,000 Da, PDI = 5.7.


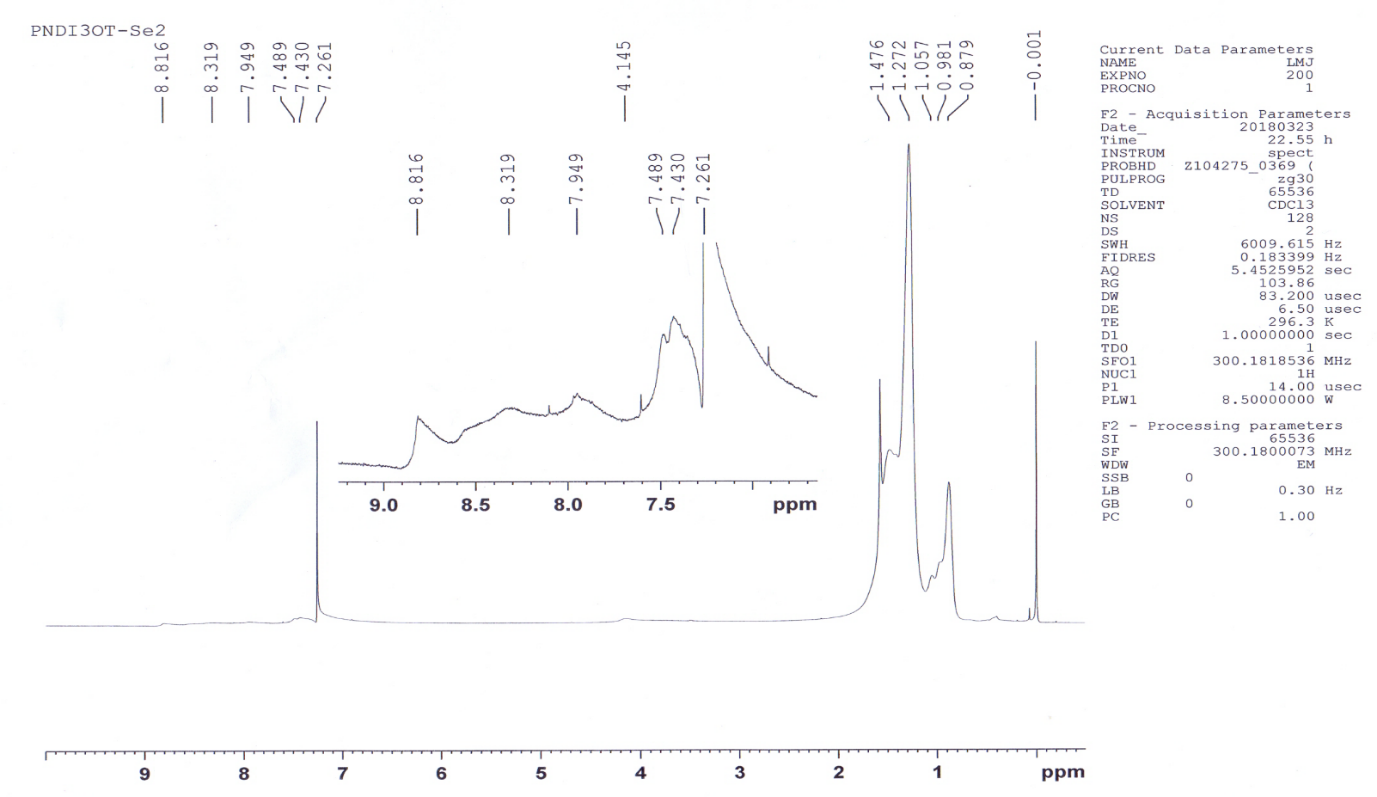


**Supplementary Figure 20** ^1^H-NMR of 477 kD P(NDI3OT-Se2).


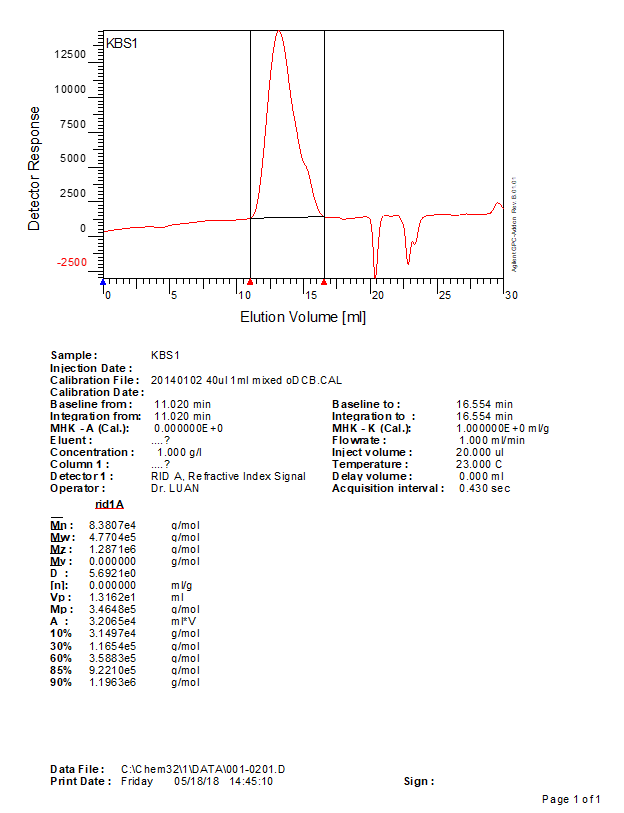


**Supplementary Figure 21** Gel permeation chromatogram of 477 kD P(NDI3OT-Se2).

**Supplymentary Figures**


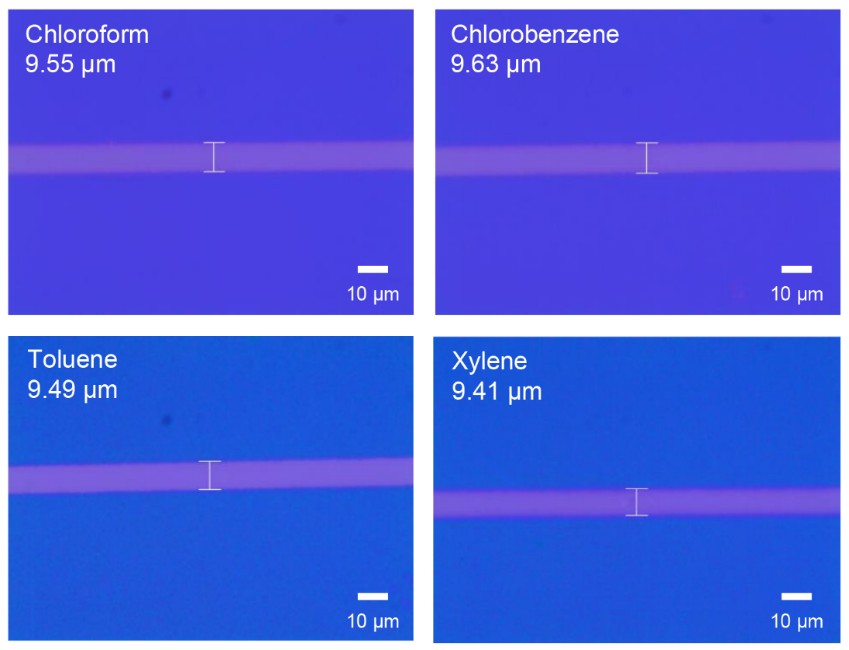


**Supplementary Figure 22** **Optical microscopy images of photopatterned P(DPP2DT-TVT) films.** These patterns were obtained by photocrosslinking P(DPP2DT-TVT) films through a photomask and removing the uncrosslinked region of the polymer using various solvents (chloroform, chlorobenzene, toluene, and xylene).


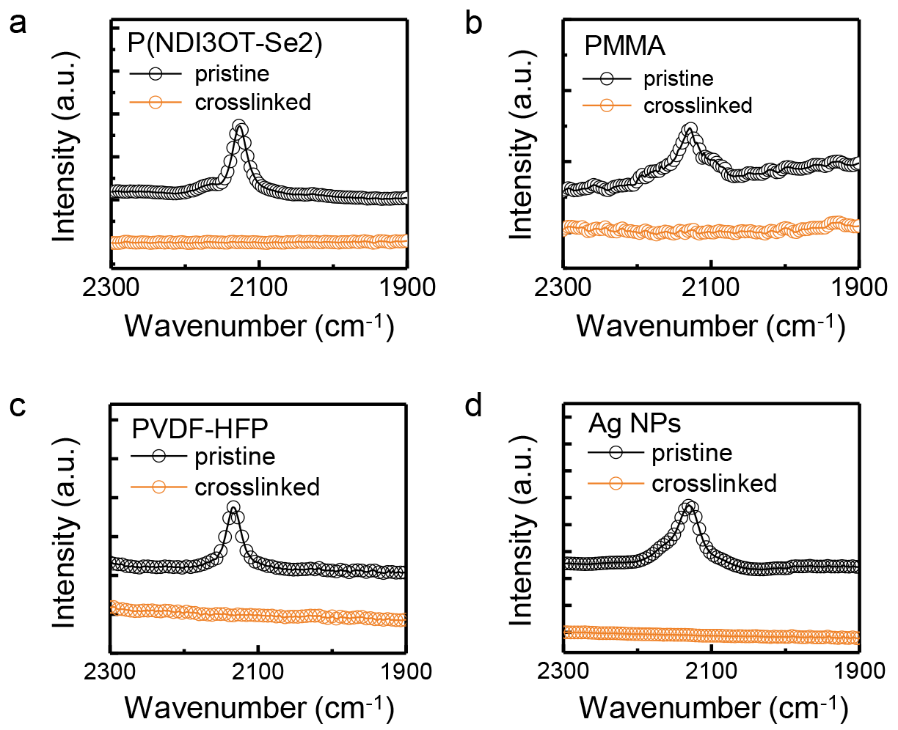


**Supplementary Figure 23** **FTIR spectra of pristine and crosslinked films** **a**, P(NDI3OT-Se2), (**b)** PMMA, (**c)** PVDF-HFP and (**d)** Ag NPs.


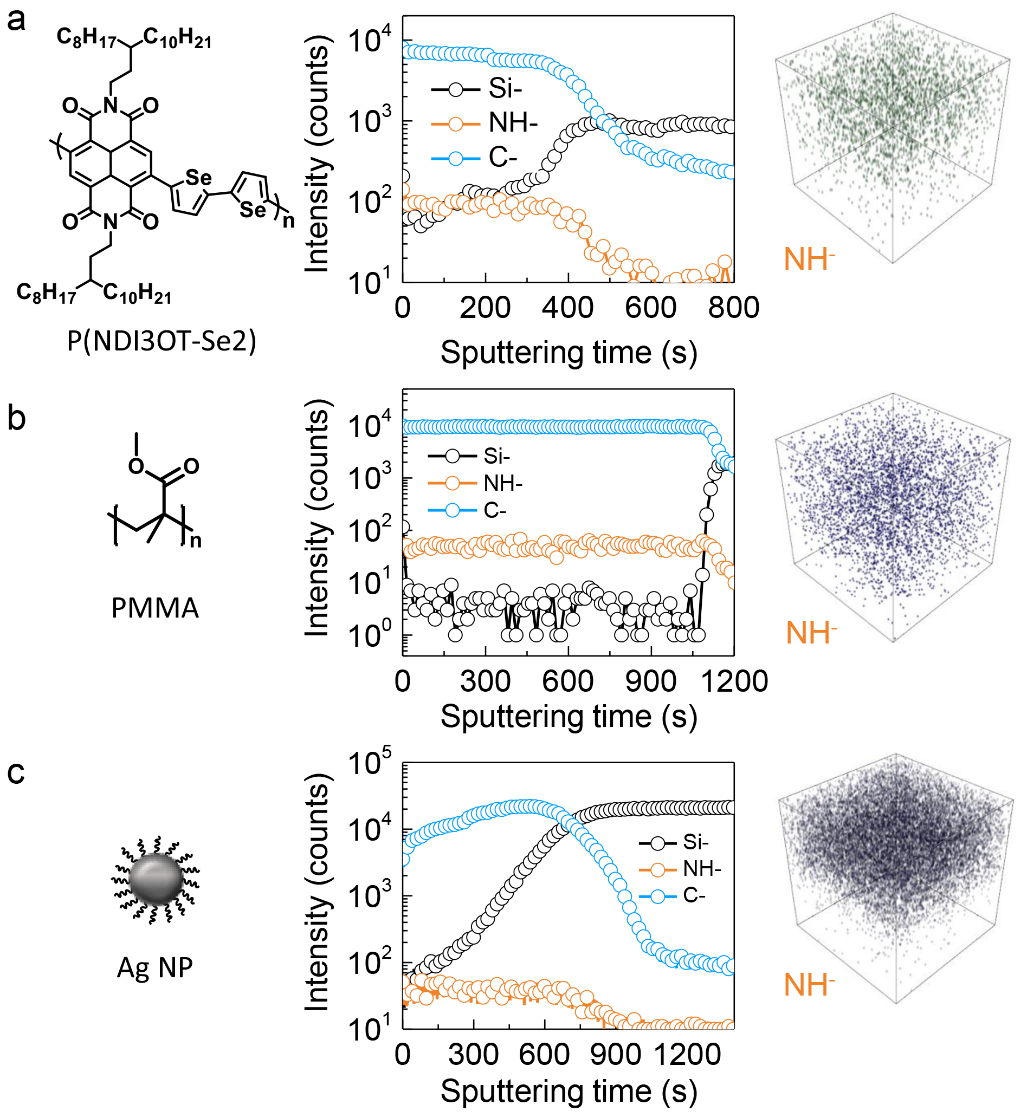


**Supplementary Figure 24 TOF-SIMS spectra of electronic component layers as a function of sputtering time** **a**, P(NDI3OT-Se2), (**b)** PMMA and (**c)** Ag NPs.


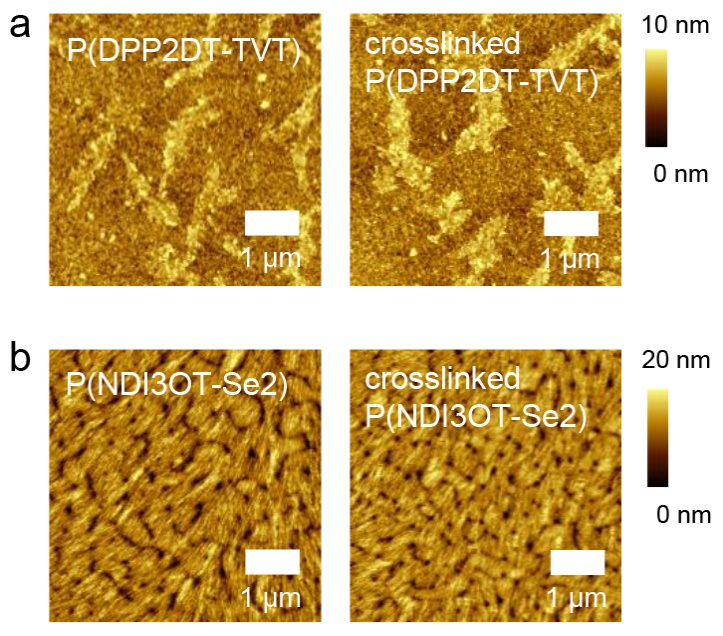


**Supplementary Figure 25 AFM topographic images of pristine and crosslinked polymer films** **a**, P(DPP2DT-TVT) and (**b)** P(NDI3OT-Se2). Crosslink was achieved using 1 wt% 4Bx under UV exposure.


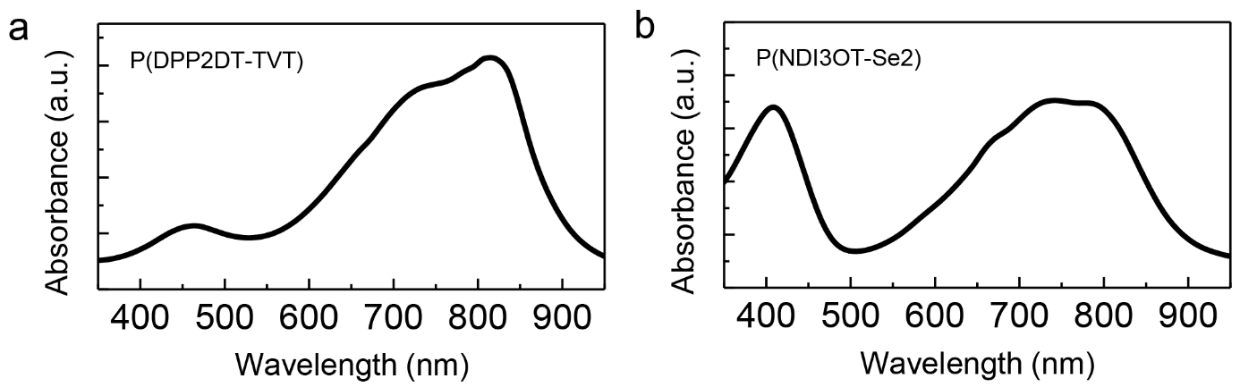


**Supplementary Figure 26 UV-visible absorption spectra** **a**, P(DPP2DT-TVT) and (**b**) P(NDI3OT-Se2) films.


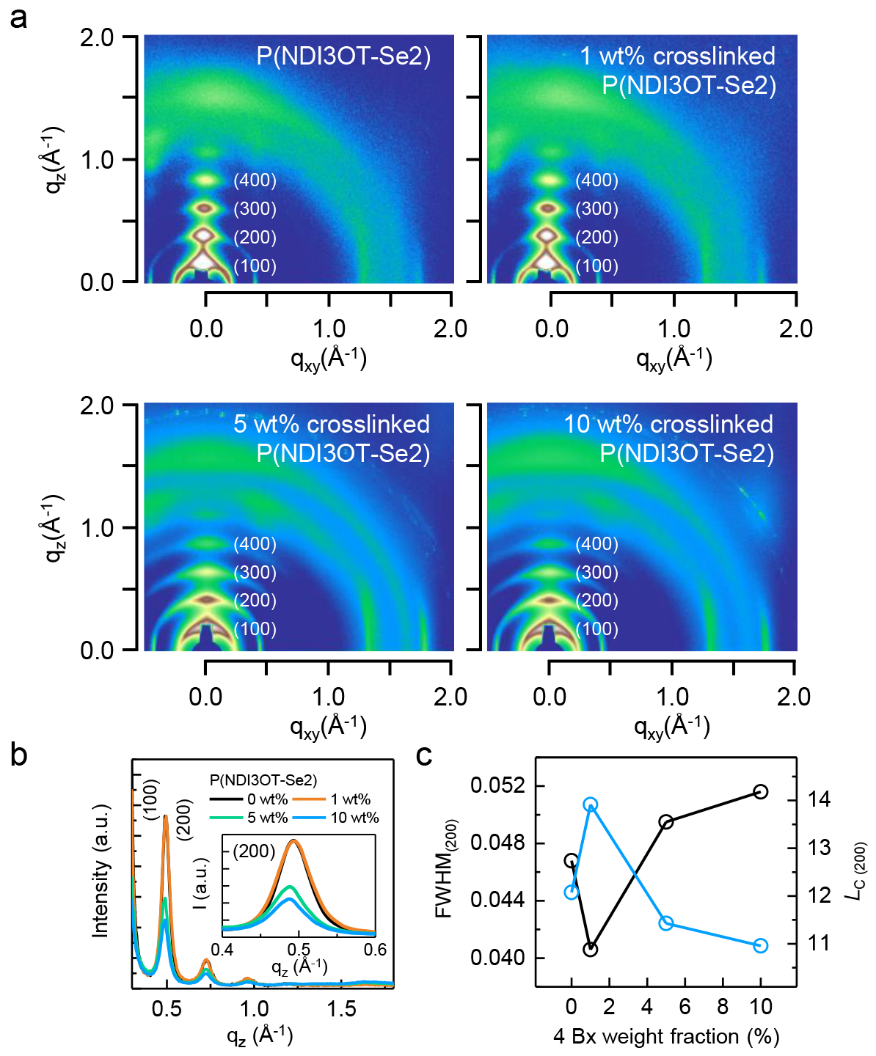


**Supplementary Figure 27** **2D GIXD analysis of crosslinked P(NDI3OT-Se2) films a**, 2D GIXD patterns of the series of P(NDI3OT-Se2) films. **b**, The line-cut diffraction profiles of out-of-plane direction of P(NDI3OT-Se2) films as function of weight fraction of crosslinker. The inset shows the enlarged (200) peaks. **c**, FWHM and *L*_C_ of the out-of-plane (200) peak of the P(NDI3OT-Se2) films as a function of weight fraction of crosslinker.


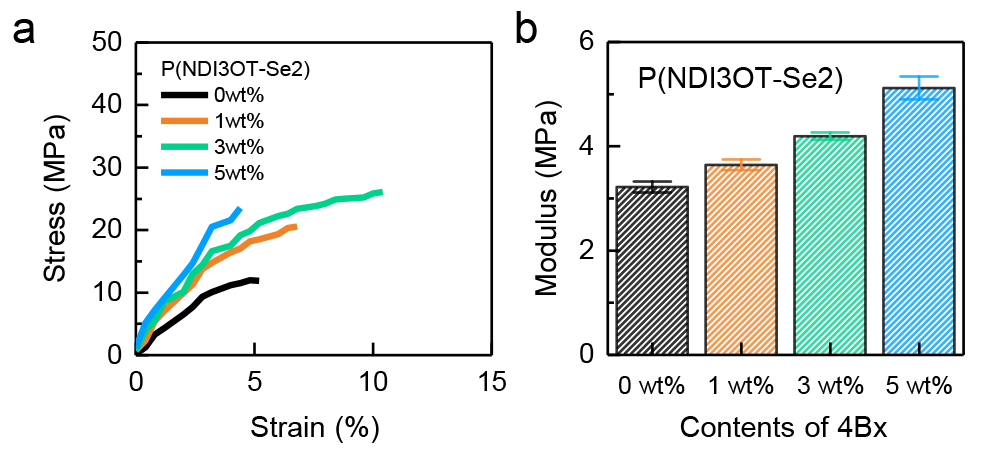


**Supplementary Figure 28 Mechanical properties of the photocrosslinked P(NDI3OT-Se2) films a**, The stress-strain curves of a series of P(NDI3OT-Se2) films crosslinked using different loadings of 4Bx (0, 1, 3, and 5 wt%). **b**, Tensile modulus of a series of P(NDI3OT-Se2) films crosslinked using different loadings of 4Bx (0, 1, 3, and 5 wt%) as measured by FOW method. The average values are obtained from 3 same samples prepared independently, and the error bars represents the standard deviation of the data.


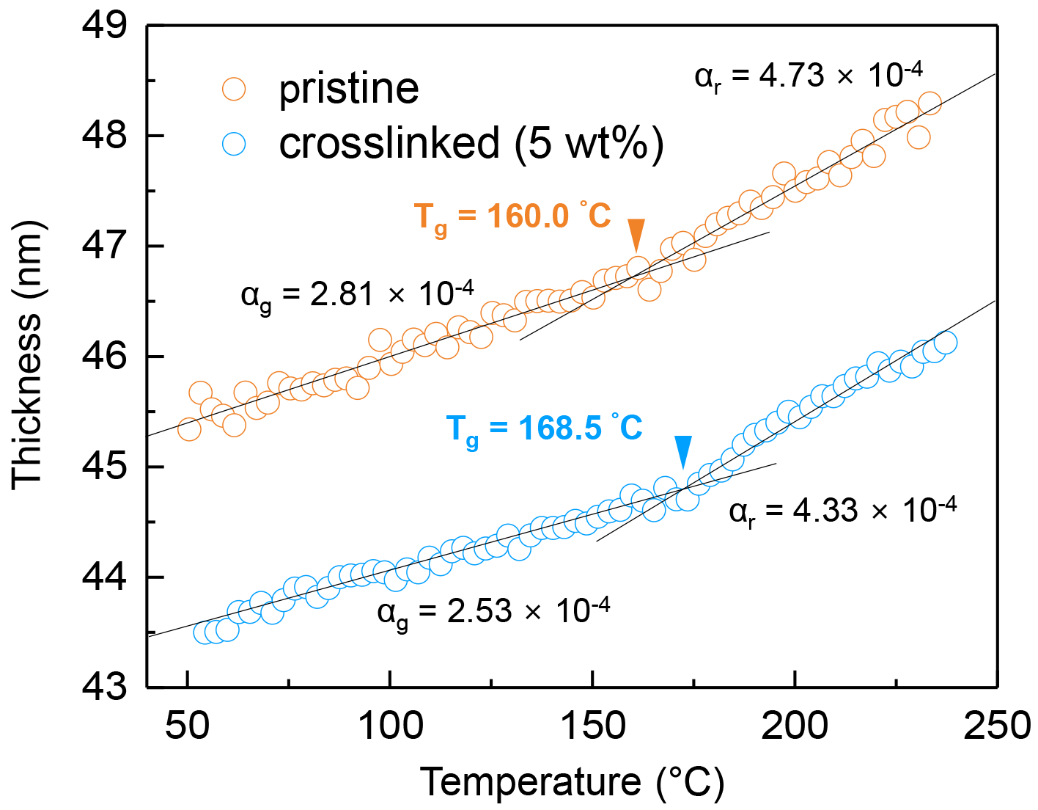


**Supplementary Figure 29 Temperature-dependent thickness measurement of P(DPP2DT-TVT) films.** Glass transition temperature (*T*_g_) of the pristine and crosslinked (5 wt%) P(DPP2DT-TVT) films.


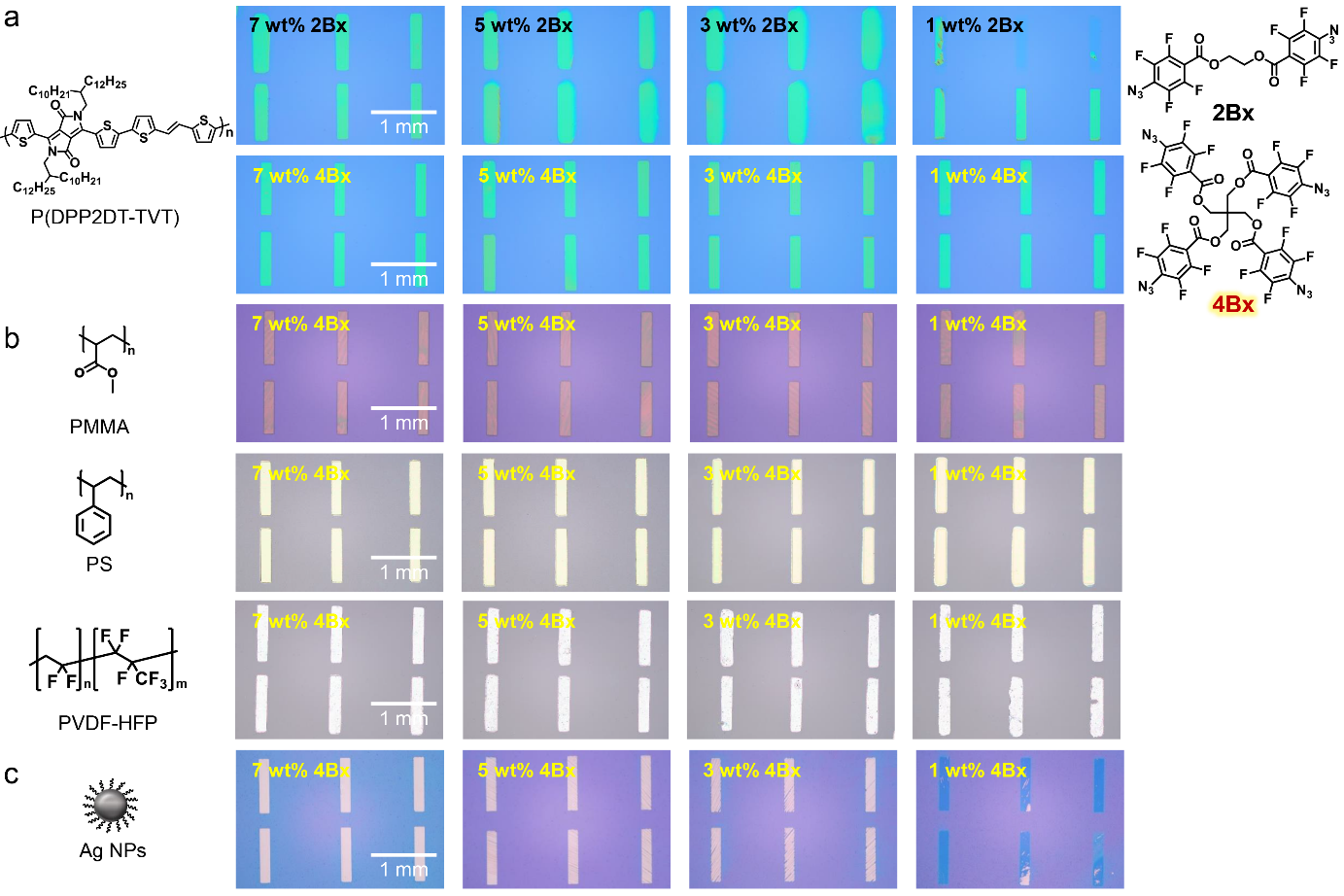


**Supplementary Figure 30 OM images of electronic component layers photopatterned using indicated amounts of crosslinker** **a**, P(DPP2DT-TVT) polymer. **b**, PMMA, PS, and PVDF-HFP polymers. **c**, Ag NPs. The chemical structures of 2Bx and 4Bx are shown on the right side of the OM images.


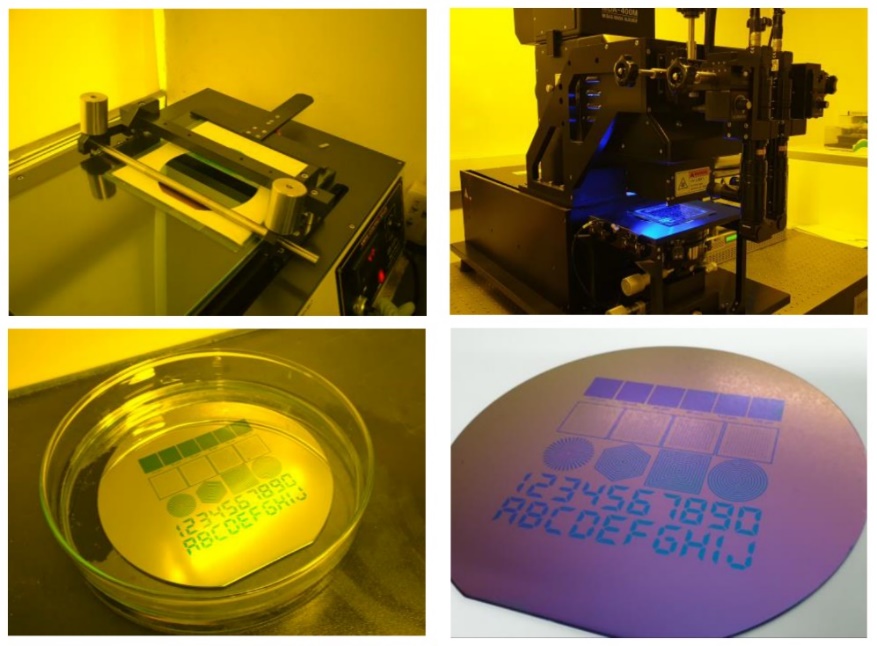


**Supplementary Figure 31 Photograph of the large-area fabrication process of photopatterned P(DPP2DT-TVT) films.** Photograph of the fabrication process of photopatterned P(DPP2DT-TVT) films on a 6-inch Si/SiO_2_ wafer. The solution for large-area patterning was prepared by adding 1 wt% of 4Bx to a solution of P(DPP2DT-TVT) in chlorobenzene (25 mg/mL). The blended solution was coated using a bar-coater (KIPAE E&T, KP-3000, Korea) onto a cleaned 6-inch Si/SiO_2_ wafer. The resulting film was exposed to a UV light through photo-mask and mask aligner (MIDAS system, MDA-400M, Korea) for 30 s. After developing the film using chloroform, patterns of P(DPP2DT-TVT) could be successfully formed.


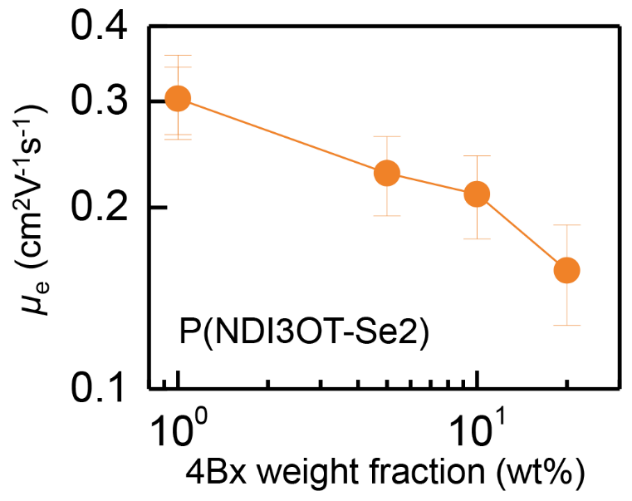


**Supplementary Figure 32** **Electron mobilities of P(NDI3OT-Se2) PTFTs.** Electron mobilities for PTFTs based on photocrosslinked P(NDI3OT-Se2) channel plotted as a function of the loading of the 4Bx. The average electron mobilities are obtained from 5 devices prepared independently, and the error bars represents the standard deviation of the data.


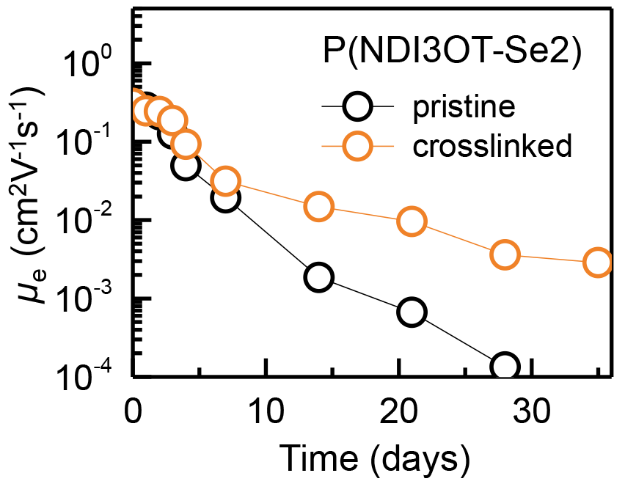


**Supplementary Figure 33 Electron mobilities of P(NDI3OT-Se2) PTFTs.** Electron mobilities are plotted as a function of exposure time to ambient conditions.


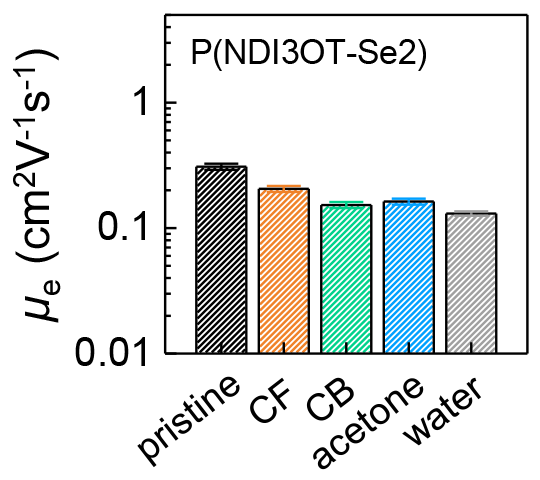


**Supplementary Figure 34 Summary of electron mobilities for photopatterned P(NDI3OT-Se2) films.** Electron mobilities of crosslinked P(NDI3OT-Se2) PTFTs before and after soaking in various solvents. The average electron mobilities are obtained from 5 devices prepared independently, and the error bars represents the standard deviation of the data.


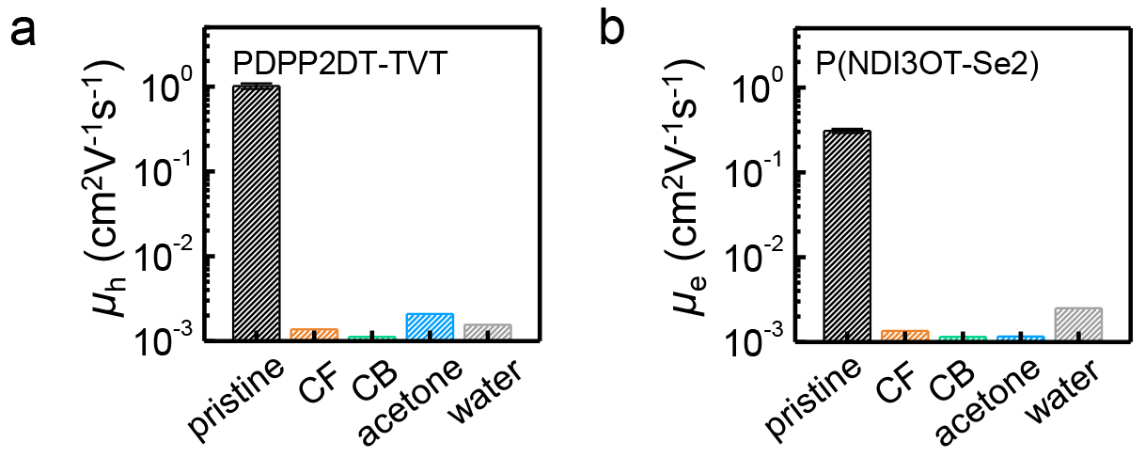


**Supplementary Figure 35 Summary of charge carrier mobilities for photopatterned polymer semiconductor films a**, Hole mobilities of pristine P(DPP2DT-TVT) PTFTs before and after soaking in various solvents. **b**, Electron mobilities of pristine P(NDI3OT-Se2) PTFTs before and after soaking in various solvents.


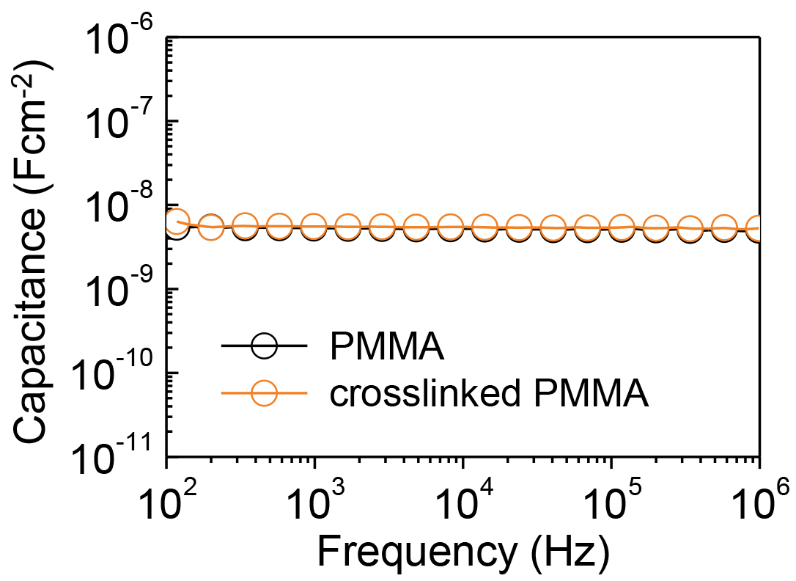


**Supplementary Figure 36** Capacitances of pristine and crosslinked PMMA dielectrics as a function of frequency.


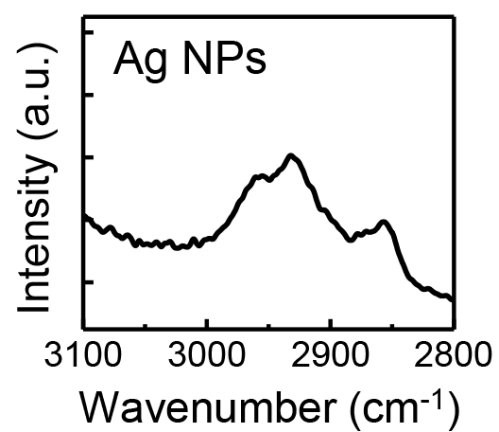


**Supplementary Figure 37** FTIR spectrum of Ag NPs containing alkyl groups as organic surfactants.


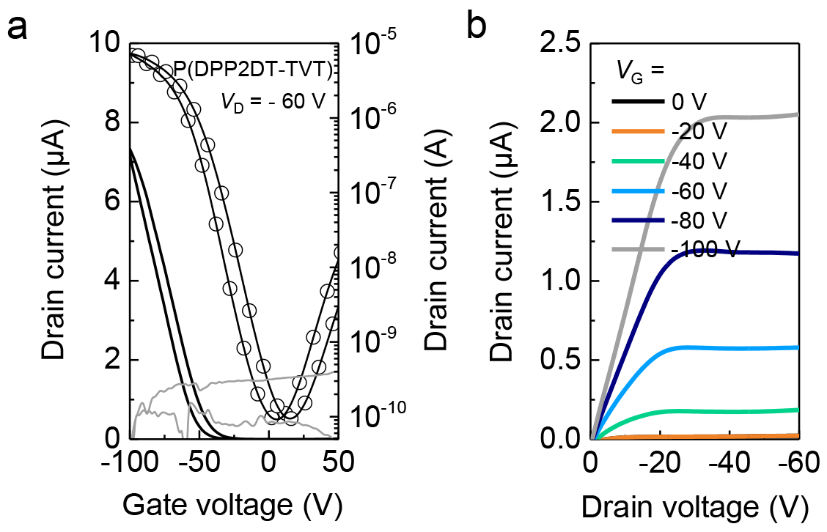


**Supplementary Figure** **38 Current-voltage characteristics** **a**, Transfer characteristics of all-solution processed, all-photopatterned PTFT fabricated in a bottom-gate top-contact (BGTC) configuration. The insets show an optical microscope image of an all-photopatterned PTFT. **b**, Output characteristics of device.


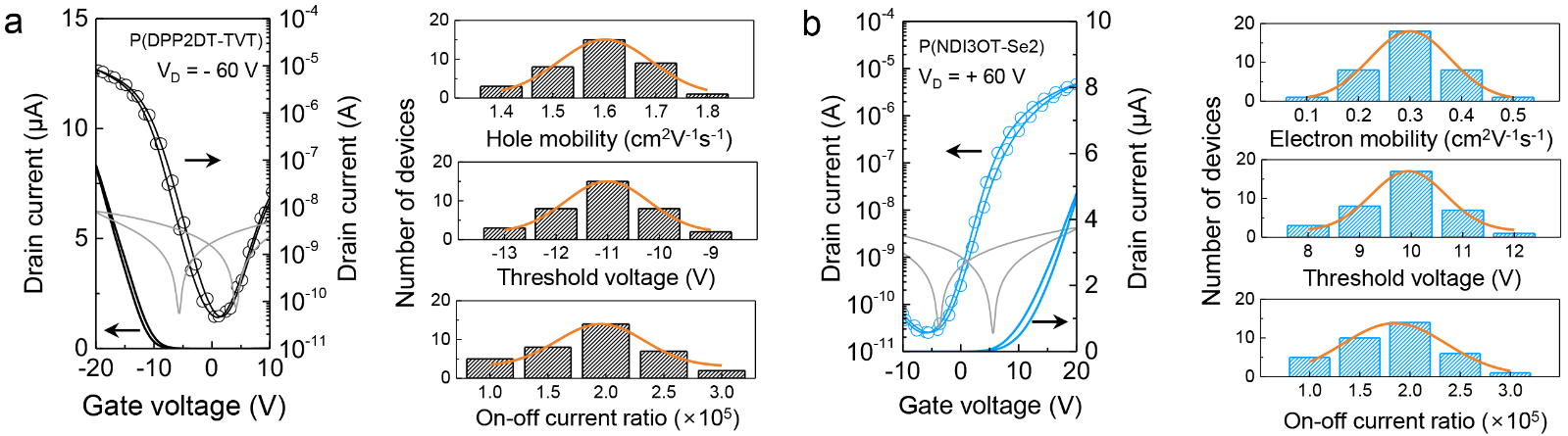


**Supplementary Fig. 39 Electrical characteristics of all-photopatterned PTFTs.** Transfer characteristics and summary of carrier mobility, threshold voltage, and on-off current ratio of (a) 36 all-photopatterned *p*-type P(DPP2DT-TVT) TFTs with PVDF-HFP gate dielectric layer and (b) 36 all-photopatterned *n*-type P(NDI3OT-Se2) TFTs with PVDF-HFP gate dielectric layer. To fabricate the gate dielectric layer, PVDF-HFP added with 5 wt% of 4Bx was dissolved together in N,N-dimethylformamide and spin-coated onto the polymer channel layer. Photocrosslinking was induced by illuminating 254 nm UV source for 30 sec.

**
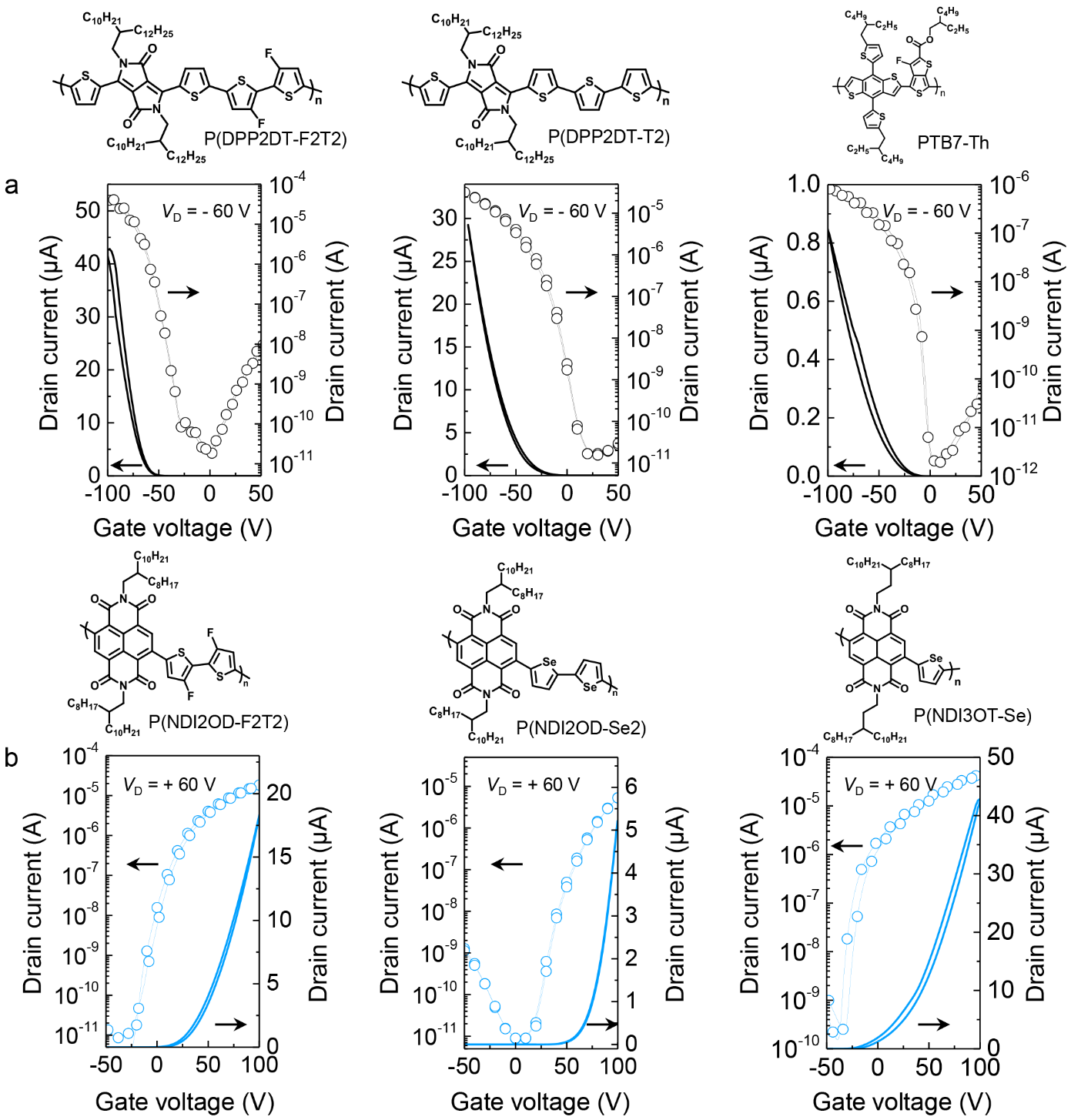
**

**Supplementary Figure 40 Transfer characteristics of all-photopatterned PTFTs a**, Chemical structures and transfer characteristics of PTFTs based on various *p*-type semiconductors: poly(2,5-bis(2-decyltetradecyl)-3-(3'',4'-difluoro-[2,2':5',2''-terthiophen]-5-yl)-6-(thiophen-2-yl)-2,5-dihydropyrrolo[3,4-*c*]pyrrole-1,4-dione) (P(DPP2DT-F2T2)), poly(3-([2,2':5',2''-terthiophen]-5-yl)-2,5-bis(2-decyltetradecyl)-6-(thiophen-2-yl)-2,5-dihydropyrrolo[3,4-*c*]pyrrole-1,4-dione) (P(DPP2DT-T2)), and poly(2-ethylhexyl 4-(4,8-bis(5-(2-ethylhexyl)thiophen-2-yl)benzo[1,2-*b*:4,5-*b*']dithiophen-2-yl)-3-fluorothieno[3,4-*b*]thiophene-2-carboxylate) (PTB7-Th). **b**, Chemical structures and transfer characteristics of PTFTs based on various *n*-type semiconductors: poly(4-(3,3'-difluoro-5'-methyl-[2,2'-bithiophen]-5-yl)-9-methyl-2,7-bis(2-octyldodecyl)-3a^1^,5a^1^-dihydrobenzo[*lmn*][3,8]phenanthroline-1,3,6,8(*2H,7H*)-tetraone) (P(NDI2OD-F2T2)), poly(4-([2,2'-biselenophen]-5-yl)-2,7-bis(2-octyldodecyl)-3a^1^,5a^1^-dihydrobenzo[*lmn*][3,8]phenanthroline-1,3,6,8(*2H,7H*)-tetraone) (P(NDI2OD-Se2)), and poly(2,7-bis(3-octyltridecyl)-4-(selenophen-2-yl)-3a^1^,5a^1^-dihydrobenzo[*lmn*][3,8]phenanthroline-1,3,6,8(*2H,7H*)-tetraone) ((P(NDI3OT-Se)).

**Supplementary Tables**

**Supplementary Table 1.** **Calculated crosslinker mole% of polymers**

| **Polymer** | **Crosslinker** | **Contents of crosslinker** (mole%) | | | |
| --- | --- | --- | --- | --- | --- |
|  |  | **1 wt %** | **3 wt%** | **5 wt%** | **10 wt%** |
| P(DPP2DT-TVT) | 2Bx | 0.43 | 1.28 | 2.14 | 4.27 |
|  | 4Bx | 0.87 | 2.59 | 4.32 | 8.65 |
| P(NDI3OT-Se2) | 2Bx | 0.45 | 1.34 | 2.23 | 4.46 |
|  | 4Bx | 0.90 | 2.71 | 4.51 | 9.02 |

**Supplementary Table 2.** **Parameters associated with the lamellar spacing**

| **Polymer** | **Crosslinker** | **Lamellar spacing from (200) in the *q*_z_ direction** | | | |
| --- | --- | --- | --- | --- | --- |
|  |  | ***q_(200)_*(Å^-1^)** | **FWHM(Å^-1^)** | ***d*(Å)^a^** | ***L*_C_(nm)** |
| P(DPP2DT-TVT) | 0 wt% | 0.513 | 0.0560 | 12.248 | 10.095 |
|  | 1 wt% | 0.517 | 0.0548 | 12.153 | 10.317 |
|  | 5 wt% | 0.517 | 0.0673 | 12.153 | 8.409 |
|  | 10 wt% | 0.513 | 0.0819 | 12.248 | 6.901 |
| P(NDI3OT-Se2) | 0 wt% | 0.489 | 0.0468 | 12.966 | 12.076 |
|  | 1 wt% | 0.488 | 0.0406 | 12.958 | 13.913 |
|  | 5 wt% | 0.486 | 0.0495 | 12.920 | 11.427 |
|  | 10 wt% | 0.486 | 0.0516 | 12.925 | 10.957 |

^a^ Lamellar spacing distance.

**Supplementary Table 3.** **Summary of device characteristics of PTFTs based on various gate dielectric polymer layers**

| Semiconductor | Dielectric layer | Dielectric constant/ Specific capacitance (nF/cm^2^) | *µ*  (cm^2^V^-1^s^-1^) | *V*_TH_  (V) | *V*_on_  (V) |
| --- | --- | --- | --- | --- | --- |
| P(DPP2DT-TVT) | PMMA | 4.7/5.0 | 0.81 (±0.18) | -56 (±4) | +7.7 (±0.5) |
|  | PVDF-HFP | 10.3/11.1 | 1.62 (±0.21) | -11 (±2) | +0.7 (±0.3) |
| P(NDI3OT-Se2) | PMMA | 4.7/5.0 | 0.15 (±0.09) | +26 (±4) | -0.6 (±0.4) |
|  | PVDF-HFP | 10.3/11.1 | 0.33 (±0.17) | +10 (±2) | -5.3 (±0.7) |

**Supplementary References**

1. Park, D. H. *et al.* Highly stretchable, high-mobility, free-standing all-organic transistors modulated by solid-state elastomer electrolytes. *Adv. Funct. Mater.* **29**, 1970116 (2019).

2. Kim, M. J. *et al.* Structure–property relationships of semiconducting polymers for flexible and durable polymer field-effect transistors. *ACS Appl. Mater. Interfaces* **9**, *40503– 40515* (2017).

3. Lee, M. *et al.* A nonchlorinated solvent-processable fluorinated planar conjugated polymer for flexible field-effect transistors. *ACS Appl. Mater. Interfaces* **9**, *28817– 28827.* (2017).

4. Jeon, G. G. *et al.* Simple solvent engineering for high-mobility and thermally robust conjugated polymer nanowire field-effect transistors. *ACS Appl. Mater. Interfaces* **10**, *29824-29830* (2018).

5. Cai, S. X., Glenn, D. J., Kanskar, M., Wybourne, M. N. & Keana, J. F. W. Development of highly efficient deep-uv and electron beam mediated cross-linkers: Synthesis and photolysis of bis(perfluorophenyl) azides. *Chem. Mater.* **6**, *1822-1829* (1994).

6. Mulherin, R. C. *et al.* Ternary photovoltaic blends incorporating an all-conjugated donor–acceptor diblock copolymer. *Nano Lett.* **11**, 4846-4851 (2011).
